# How do aspects of selfhood relate to depression and anxiety among youth? A meta-analysis

**DOI:** 10.1017/S0033291723001083

**Published:** 2023-08

**Authors:** GeckHong Yeo, Cameron Tan, Dean Ho, Roy F. Baumeister

**Affiliations:** 1 N.1 Institute for Health, National University of Singapore, Singapore; 2 The Institute for Digital Medicine (WisDM), Yong Loo Lin School of Medicine, National University of Singapore, Singapore; 3 Department of Biomedical Engineering, NUS Engineering, National University of Singapore, Singapore; 4 Department of Pharmacology, Yong Loo Lin School of Medicine, National University of Singapore, Singapore; 5 School of Psychology, University of Queensland, Brisbane, QLD, Australia

**Keywords:** Adolescent, anxiety, depression, mental health, meta-analysis, selfhood

## Abstract

Adolescents' sense of self has important implications for their mental health. Despite more than two decades of work, scholars have yet to amass evidence across studies to elucidate the role of selfhood in the mental health of adolescents. Underpinned by the conceptual model of selfhood, this meta-analytic review investigated the strength of associations of different facets of selfhood and their associated traits with depression and anxiety, moderating factors that attenuate or exacerbate these associations, and their causal influences. Using mixed-effects modeling, which included 558 effect sizes from 298 studies and 274 370 adolescents from 39 countries, our findings revealed that adolescents' self-esteem/self-concept [*r* = −0.518, *p* < 0.0001; (95% CI −0.49 to −0.547)] and self-compassion [*r* = −0.455, *p* < 0.0001; (95% CI −0.568 to −0.343)] demonstrating largest effect sizes in their associations with depression. Self-esteem/self-concept, self-compassion, self-awareness, self-efficacy, and self-regulation had similar moderate negative associations with anxiety. Meta-regressions revealed that adolescent age and type of informants (parents *v.* adolescents) were important moderators. Findings on causal influences indicated bidirectional causations, particularly low self-esteem/self-concept, self-awareness and self-efficacy drive higher depression and vice-versa. In contrast, the different self traits did not demonstrate specific causal direction with anxiety. These results pinpoint self traits that are pivotal in relating to adolescent mental health functioning. We discussed the theoretical implications of our findings in terms of how they advance theory of selfhood for adolescent mental health, and the practical implications of building selfhood as cultivating psychological skills for mental health.

Mental health problems increase during adolescence, with depression and anxiety becoming more common than they were during childhood (Wellcome Trust, 2021). Adolescence is also a period of profound biological, social, and emotional changes that occur in the formation of the adult self (e.g. Erikson, 1950, 1968), and underscores selfhood development – the experience of negotiating the self that entails a dynamic, causally connected system of thoughts, feelings, and behaviors. This unity of inner processes and their coordinated functioning allow individuals to interact and function effectively in social contexts (Baumeister, 1999, 2022). Cognitive advancement involving introspection and integration of schemas facilitate the development of self-awareness and coherent self-concept among adolescents (Weil et al., 2013). Adolescents' heightened attempts at establishing and evaluating a stock of knowledge about the self increase their sensitivity to affective cues and experiences of emotional fluctuations, which lead to greater propensity for anxiety and depression (Derosa, 2000; Luyten & Fonagy, 2016).

At the same time, self and identity development become crucial as adolescents establish and maintain more complex social relationships, including peer and romantic relationships (Brown & Larson, 2009). In developing these relationships, adolescents' concern about social comparison and impression management can result in the experience of internalizing symptoms that often manifest as unease, tension, and suffering (Levesque, 2011; Zahn-Waxler, Klimes-Dougan, & Slattery, 2000). The compounding of these symptoms leads to syndromes such as depression and anxiety (Levesque, 2011; Zahn-Waxler et al., 2000). Adolescence is also a time when individuals achieve greater individuation and agency (Zimmer-Gembeck & Collins, 2003). Mastery beliefs and competency in regulating thoughts, behaviors and emotions have immediate and prospective implications for adolescents' development of psychopathology (Bakker, Ormel, Verhulst, & Oldehinkel, 2011; Rhodes et al., 2013). Hence, navigating key developments of the self increases adolescents' susceptibility to mental health conditions (WHO, 2021).

In addressing youth mental health as a pressing public health need (WHO, 2021), recent efforts have focused on evidence-based practices in cultivating a set of psychological skills surrounding the self to enhance mental health functioning (Dahl, Wilson-Mendenhall, & Davidson, 2020). Despite the voluminous research on selfhood and adolescent psychopathology, findings on their relations remain scattered. Studies rarely refer to or feature a conceptual framework that delineates selfhood development in relation to mental health conditions (de Jong, Sportel, de Hullu, & Nauta, 2012; Gittins & Hunt, 2020). Existing work suggests that adolescents' age and perception (*v.* parental perception) modulate the link of selfhood and mental health (Herman, Ostrander, & Tucker, 2007; Watson, 2006). Finally, the casual direction between adolescent selfhood and mental health is not clear (de Jong et al., 2012; Gittins & Hunt, 2020), and research efforts at synthesizing findings from longitudinal investigations can elucidate the directionality of influence (e.g. Wang, 2011; Zhou, Li, Tian, & Huebner, 2020).

A review of studies amassing evidence on the relation of selfhood and adolescent psychopathology that is guided by a conceptual model is needed (Choi & Choi, 2016; Gardner & Lambert, 2019). A nuanced and comprehensive understanding of selfhood and adolescent mental health could set the stage for theoretical refinements and improvements to intervention efforts. This review may inform future research as well as evidence-informed practices for professionals such as education stakeholders aiming at adolescents' selfhood development cultivate selfhood as psychological skills of mental well-being (Dahl et al., 2020). The present work addresses the need for a review of literature on selfhood and adolescent mental health implications by comparing how different facets of the self and their associated traits relate to specific form of psychopathology. Using a meta-analytic approach, we undertook to test the relationships of different self traits with two most prevalent mental health conditions in adolescence – depression and anxiety (de Jong et al., 2012; Eisenberg, Gollust, Golberstein, & Hefner, 2007) – in three ways. First, we established what self traits are most clearly and strongly linked to depression and anxiety. Second, we elucidated individual factors that amplify or attenuate the selfhood-psychopathology linkages. Third, we examined the direction of causality: Are problems and deficiencies in selfhood the cause, the result, or merely a correlate of depression and anxiety?

Our review and meta-analytic approaches were organized based on the tripartite model of selfhood (Baumeister, 1999, 2022). The self can be conceptualized in terms of three functioning parts: reflexive awareness (self-knowledge), interpersonal being, and agentic executive function (Baumeister, 2022). Reflexive awareness refers to the basic awareness of self and the accumulated set of beliefs and concepts about oneself. In terms of relevant traits, these includes self-esteem (e.g. Barry, Loflin, & Doucette, 2015; Stolow, 2016), self-compassion (Neff, 2011), self-concept, and basic or private self-awareness (e.g. Chang, 2001; Hards, Ellis, Fisk, & Reynolds, 2020). Interpersonal self involves the self that is formed in social interactions and relationships and fewer traits seem relevant, especially in terms of what is available in the literature. A major and most widely examined interpersonal self trait would be public self-consciousness (also known as public self-awareness) – the tendency to think and care deeply about how one is perceived by others (Fenigstein, Scheier, & Buss, 1975). Third, the agentic self involves how the self takes action to manage its affairs. Self-regulation is a prominent and influential aspect of this, and it encompasses how the self operates to alter its responses, especially toward improving long-term goals (e.g. Moffitt et al., 2011). Self-efficacy is another relevant trait; it refers to how well the individual assesses his or her ability to perform the optimal responses in a given situation (Bandura, 1977).

## How does selfhood relate to adolescent depression and anxiety?

Based on the tripartite model of selfhood, the broad notion is that a more developed self would go hand in hand with better mental health (Baumeister, 1999, 2022), that is, the higher the person scored on self-esteem, self-concept, self-compassion, self-awareness, self-efficacy, and self-regulation the lower the person's depression and anxiety would be (Baumeister, 1999, 2022). However, the relation of selfhood and adolescent mental health is much more nuanced and complex, as the various self traits can have differential relations with different forms of psychopathology (Harter, 2012; Luyten & Fonagy, 2016). Our review of the literature revealed that studies have predominantly focused on the facet of self-knowledge in relation to adolescent psychopathology, which includes the traits of self-esteem, self-concept, self-compassion, and (private) self-awareness (e.g. Hards et al. 2020; Surdey, 2016), and particularly self-esteem (e.g. Barry et al., 2015; Stolow, 2016) and self-compassion (Neff, 2011).

### Reflexive awareness (self-knowledge)

Fourteen out of the 298 studies included in our meta-analysis examined self-esteem, self-compassion, self-concept and self-awareness conjointly and found that they are related but distinct, with low to moderate associations (−0.11 < *r* < 0.71; e.g., Barry et al. 2015; Stolow, 2016). Findings are mixed in how self-esteem, self-compassion, self-concept, and self-awareness relate to adolescent mental health (e.g. Chang, 2001; Weber, Puskar, & Ren, 2010), particularly with depressive and anxiety symptoms (e.g. Grøholt, Ekeberg, Wichstrøm, & Haldorsen, 2005; Schwartz et al., 2006). Particularly for self-esteem, the longstanding and overstated view of low self-worth contributing to poor mental health functioning has been challenged on several instances. Scholars have noted a much more complex role of self-esteem, particularly how fragile self-esteem, pursuit of high self-esteem, and overly high self-esteem can be detrimental to adolescent mental health functioning (Luyten & Fonagy, 2016; Rosenfield, Lennon, & White, 2005). The literature also indicates mixed predictions involving self-awareness. Ingram (1990) reviewed multiple links between high self-awareness and greater mental illness. On the other hand, studies found that greater self-awareness confers benefits for adolescent mental health (e.g. Chang, 2001; Weber et al., 2010), particularly lower depressive and anxiety symptoms (e.g. Grøholt et al., 2005; Schwartz et al., 2006). Given that negative cognitions and feelings about the self typically characterize depressed individuals, self-compassion may play a specific role in and confer benefits for depression, but less so for anxiety (Baumeister, 2022; Neff, 2011).

### Interpersonal self

There is comparatively less research attention on adolescents' interpersonal self in general and its relation to mental health in specific (e.g. Arrington, 2001; Bowker & Rubin, 2009). Theoretical and empirical work have demonstrated that the interpersonal self has low to moderate positive associations with and is not analogous to self-knowledge and self-agency (Derosa, 2000; Wright, 2005), which suggests that the interpersonal self has distinct relation to adolescent mental health. Because the interpersonal self is embedded in relationships, a major trait that is most widely examined is public self-consciousness (also known as public self-awareness; Fenigstein et al., 1975). The importance of public self-consciousness in adolescence is well-recognized (Jackson, 2007; Wang et al., 2017), especially noting how making a good impression among peers, gaining status in the peer group, and peer popularity can heighten adolescents' sensitivity to affective cues (Jackson, 2007; Sebokova & Popelkova, 2016). Establishing and maintaining more complex social relationships in adolescence require individuals to pay attention to how they are regarded by others (Brown & Larson, 2009; Hards et al., 2020), which can intensify emotional lability and increase susceptibility to depressive and anxiety symptoms (Fenigstein et al., 1975; Sebokova & Popelkova, 2016).

### Agentic self

Adolescents' self-agency and the associated traits of self-regulation and self- efficacy are distinguished from and have low positive associations with self-knowledge and interpersonal self (0.14 < *r* < 0.75; e.g. Cañas, Estévez, Estévez, & Aparisi, 2020; Muris et al., 2017). Developmentally, self-regulation and self-efficacy become more salient because of greater agency in making choices and exerting control in adolescence (Zimmer-Gembeck & Collins, 2003). With biological changes and increased significance of social relations, adolescents are more sensitive to emotional cues and are more prone to risk-taking behaviors (Steinberg, 2005, 2010), which underscore the need for self-regulation and self-efficacy abilities (Rhodes et al., 2013; Wright, 2005). Adolescents' self-efficacy and self-regulation have immediate and prospective mental health implications (e.g. Caprara, Gerbino, Paciello, Di Giunta, & Pastorelli, 2010; Rhodes et al., 2013), and often often mediate psychological mechanisms leading to depression and anxiety (e.g. Loton & Waters, 2017; Romera, Gómez-Ortiz, & Ortega-Ruiz, 2016).

Taken together, our review found that research has rarely considered the conceptual framework of selfhood in understanding adolescent mental health (e.g. Stormshak, DeGarmo, Chronister, & Caruthers, 2018; Wasylkiw, MacKinnon, & MacLellan, 2012). Research attention is warranted in distinguishing the relations of different facets of selfhood and their associated with specific form of psychopathology among young people. Synthesizing studies that investigated different traits of self-knowledge, interpersonal self and self-agency make important theoretical contributions – specifically, aspects of the self that are common across different forms of psychopathology, and those that are unique to specific form of psychopathology (Baumeister, 2022). Work such as this can inform interventions selfhood development as psychological skills adolescents can cultivate to benefit their mental health (Dahl et al., 2020).

## What individual factors moderate the links of selfhood to depression and anxiety?

Developmental trends in adolescent selfhood and depression and anxiety, as well as adolescent perspective (*v.* parental perspective) are potential individual factors that moderate the link between selfhood and mental health functioning (e.g. Miyamoto et al. 2001; Murray, Rieger, & Byrne, 2015). Meta-analyses and reviews found that self-esteem increases as adolescents grow older (e.g. Bachman, O'Malley, Freedman-Doan, Trzesniewski, & Donnellan, 2011), but developmental trends in other self traits are less clear (Bluth, Campo, Futch, & Gaylord, 2017; Onetti, Fernández-García, & Castillo-Rodríguez, 2019). In early adolescence, individuals experience greater emotional lability and have less effective coping strategies, which can give rise to the experience of greater depressive and anxiety symptoms (Liu, Jiang, Li, & Yang, 2021; Schwartz et al., 2006). Additionally, the relation of selfhood and mental health may be particularly salient among younger adolescents because of biological changes such as puberty, a less stable and coherent sense of identity, and individuation from parents (de Jong et al., 2012; Harter, 2012) – a notion that has not been subjected to empirical examination.

Studies that investigated adolescent selfhood and its relation to depressive and anxiety symptoms have documented differences in adolescent and parental perceptions (DuBois, Bull, Sherman, & Roberts, 1998; DuBois, Felner, Brand, & Phillips, 1996). There is substantial work demonstrating that parental reports of adolescent psychopathology are heavily influenced by the parent's own mental health (Hope et al., 1999; Stokes, Pogge, Wecksell, & Zaccario, 2011). It is reasonable to argue that adolescents are the best judges of their selfhood development. Parents' reports more often reflect the extent to which they believe the child possesses self-knowledge, interpersonal relationships, and agency, instead of an objective report on the child's selfhood (Smith, 2007; as might be argued for parents' reports of the child's psychopathology). Thus, it seems logical and more appropriate to rely mainly on adolescents' reports of their own symptoms and self traits. However, assessing reporter effects (parent and child) provides important insights because the absence of informant effects would suggest that although parents cannot report accurately on their adolescent-child selfhood development, they may rely on additional sources of accurate information beyond the child, such as siblings, peers, and teachers whom the adolescents interact with (Hope et al., 1999; Stokes et al., 2011). Elucidating the moderating effects of parental and adolescent perceptions can indicate the need for future investigations of adolescent selfhood development and psychopathology to proceed from a broader frame of reference.

## What is the causal direction of selfhood with depression and anxiety – specifically, does selfhood concurrently relate to, drive, or result from depression and anxiety?

Directionality was difficult to infer: It could be that mental illness causes one to have less intra- and interpersonal capacity and resources for developing the self (Selye, 1950). Alternatively, it could be that a less developed self has preoccupation with self deficiencies instead of strengths (Luyten & Fonagy, 2016). Inner ruminations may draw attention to one's problems and thereby enhance them, as compared with focusing attention outward on the social environment, including other people, relationships, and external problems (Luyten & Fonagy, 2016). More broadly, competing hypotheses can be examined regarding all possible causal relationships. Selye's (1950) theory of a General Adaptation Syndrome proposes that mental health influences selfhood development. This occurs at least partly because mental illness symptoms reduce intra- and interpersonal capacities and resources that are needed for healthy development of the self. For instance, mentally well-functioning individuals are better able to introspect and gain self-awareness (Sutton, 2016), build an integrated concept of the self (Orchard, Westbrook, & Gee, 2021), and form positively biased self-evaluation (Owens, 1993). In contrast, many theories propose that selfhood development is a main causal driver of mental illness. As examples, self-determination (Sheldon, 2012) and self-reliance schema theories (Rosenfield et al., 2005) argued that the development of the self is pivotal for navigating developmental tasks and life challenges (Sheldon, 2012), understanding one's strengths, and forming boundaries that separate the self from others (Rosenfield et al., 2005) – precursors to positive mental health functioning.

A third perspective would emphasize bidirectional causation: selfhood development and mental health influence each other reciprocally. The training-based framework of mental well-being would support bidirectional causation between selfhood and mental health functioning (Dahl et al., 2020). This perspective posits, for instance, that when individuals cultivate self-awareness, they develop the ability to introspect and establish both positive and negative cognitions. This balanced state that entails equal access to both positive and negative cognitions is important in producing and maintaining positive mental health (e.g. Beck, 1967) (In fact, a slightly more positive cognitive state seems to characterize healthy mental functioning; Baumeister, 2022). On the other hand, one's mental state can drive self-awareness by affecting the relative access to positive and negative cognitions (Teasdale & Fogarty, 1979). Thus, self-awareness (or lack thereof) can produce psychopathology, and conversely, psychopathology can increase cognitive vulnerability, particularly heightened awareness of or access to negative cognitions that further perpetuate psychopathology. Supporting the notion of bidirectional causation, theories and empirical findings demonstrated the reciprocal interplay of depression and negatively biased self-awareness that forms a perpetuating cycle that leads to greater depression (Teasdale, 1983).

## The current study

Despite the voluminous evidence on selfhood and the link to adolescent mental health, particularly depression and anxiety, three important gaps remain. First, studies that examined self traits in relation to depression and anxiety among adolescents are scattered (de Jong et al., 2012; Gittins & Hunt, 2020), and there is no existing review that synthesizes findings to compare how different self traits differ in their relations to the two mental health conditions. Second, findings on the moderating role of individual factors involving adolescent age and perception are mixed (Herman et al., 2007; Watson, 2006), and it is not clear how these factors attenuate or amplify the selfhood-mental health links. Finally, accumulating findings from longitudinal studies emphasize the need to elucidate the causal linkages of adolescent selfhood with depression and anxiety (e.g. Wang, 2011; Zhou et al. 2020). Towards this end, our meta-analysis examined the following research questions (RQ)

- **RQ1: How does selfhood relate to adolescent depression and anxiety?**
- **RQ2: What individual factors moderate the links of selfhood to depression and anxiety?**
- **RQ3: What is the causal direction of selfhood with depression and anxiety – specifically, does selfhood concurrently relate to, drive, or result from depression and anxiety?**

## Method

This meta-analysis was conducted based on the guidelines provided by Quintana (2015) and reported in accordance with the latest version of Preferred Reporting Items for Reviews and Meta-Analyses guidelines (Page et al., 2021). A protocol was registered a priori (PROSPERO registration number CRD (Blinded)).

### Literature search

With the assistance of a staff librarian at the first author's affiliated institution, two research assistants independently employed three search strategies to systematically collect empirical studies on adolescent selfhood and mental health. First, a systematic search across EBSCOhost and Pro-Quest was conducted April 2021 and updated again June 2022 to include articles published from 1 January 2000 through June 2022. The starting date was chosen based on the publication of Baumeister's (1999) conceptual model of selfhood. Searches were re-run just before the final analyses and any further studies identified were retrieved for inclusion. EBSCOhost and Pro-Quest were selected because they cover two fields pertinent to this review: social science and medical research. They subsume 93 online databases, including PsycINFO, Cumulative Index to Nursing and Allied Health (CINAHL), PubMed, and EMBASE. Second, reference lists of the included studies were searched manually and cross-referenced for additional articles. Third, we manually searched in-press or online-first article abstracts in the following journals: *Psychological Medicine, Child Development, Developmental Psychology, Journal of Adolescence, Journal of Child and Family Studies, Journal of Family Psychology, Journal of Research on Adolescence, Journal of Social and Personal Relationships, Journal of Youth and Adolescence,* and *Social Development.* Together, these search methods identified 71 614 citations to relevant studies. Table 1 details the search terms that were developed using the PICO (Population Intervention Comparison Outcome) search strategy. Full search strings are provided in the Appendix (online Table A1).

**Table 1.** Search terms used for the formulation of the search strings in the systematic search

PICO concept	Search terms
Population	Adolescents, youth, young person, young people, teenager (10–18 years) and emerging adults (19–25 years)
Intervention (or Exposure)	self-esteem, self-worth, self-regard, self-evaluation, self-compassion, self-blame, self-acceptance, self-concept, self-understanding, self-description, self-identity, self-awareness, self-image, self-liking, self-confidence, self criticism, self-efficacy, self-regulation, emotional regulation, self-control, self-autonomy, self determination, self-consciousness, self-competence, self-presentation
Comparison	None
Outcome	Anxiety, depression, mental well-being, psychological well-being, distress, loneliness, internalizing symptom*, externalizing symptom*, internalizing symptom*, externalizing symptom

*Note.* Included both UK and US spelling for all the search terms listed.

### Study selection

Study screening was conducted using Covidence software (Veritas Health Innovation, Melbourne, Australia). Articles were independently screened first by title and abstract by two reviewers (undergraduate research interns). All articles selected for full text review were independently screened by another two reviewers (first author and an undergraduate research intern) with any queries and discrepancies resolved through discussion. A third reviewer was contacted if consensus could not be reached. Inclusion and exclusion criteria were established *a priori*. To be included, an article needed to:

- be a full-text empirical study written in English and published between 2000 and 2021;
- feature early to late adolescents aged 10 to 25 and/or their parents (note that parents were not rating themselves but their adolescent children);
- provide sufficient statistics for calculation of effect sizes;
- include at least one measure of selfhood;
- include at least one measure of depression and anxiety (refer to Table 1 for the full list of search terms).

**Figure 1.**
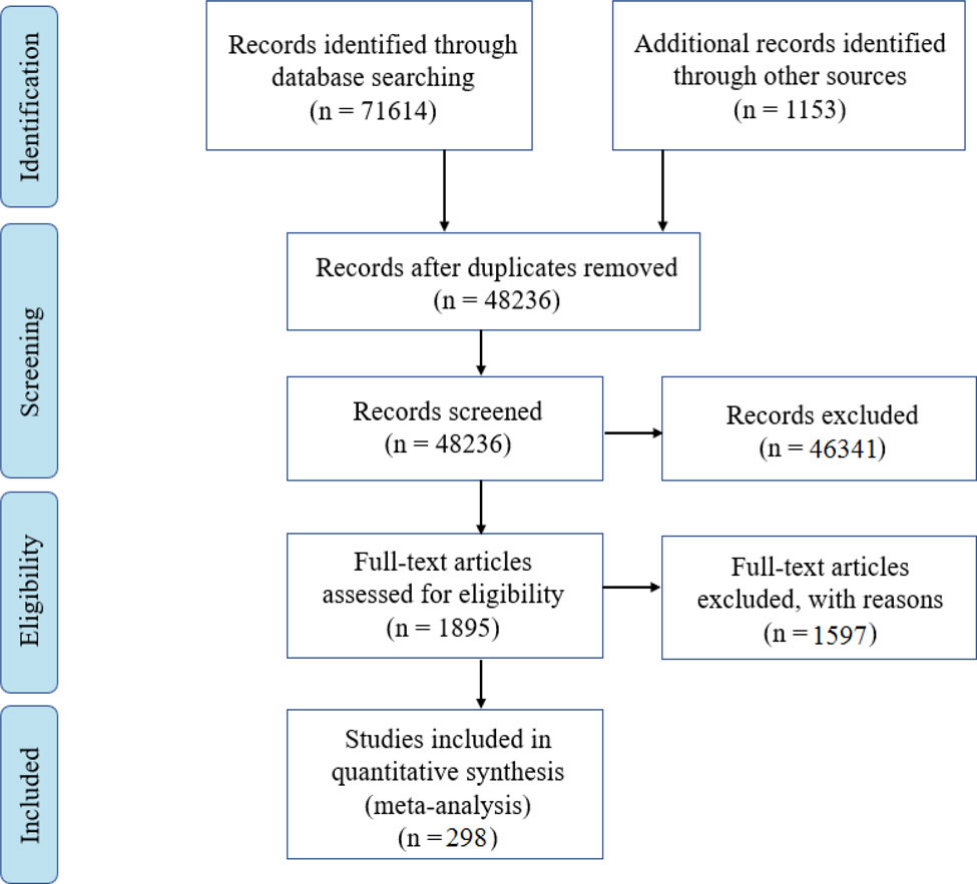
PRISMA Flow diagram of search strategy on identification and screening of studies, and the finalized number of articles for meta-analysis.

Of the 72 767 relevant studies identified in the initial searches, 24 531 were duplicates. First stage screening of the 48 236 remaining articles entailed checking of titles and abstracts, which led to the elimination of 46 341 studies. The remaining 1895 full-text articles were assessed for eligibility. 415 investigations had samples with the wrong populations – average ages outside the specified age range; 535 studies had wrong factors and study designs (e.g. information that did not concern the six self traits; 139 studies could not be retrieved; 19 were duplicates; 200 examined outcomes that did not concern depression and anxiety. An additional 289 did not report sufficient statistics required to compute effect sizes (authors of these studies failed to respond to requests for the needed information). 298 studies with 389 effect sizes on selfhood-depression links and 169 effect sizes on selfhood-anxiety links were included in our meta-analyses. Figure 1 presents a flowchart of the study selection process.

### Data extraction and coding

Two undergraduate research assistants independently extracted and coded data on study design, sample characteristics, mental health outcomes, concept definitions and operationalizations (six self traits), Cronbach alphas, and correlation coefficients (see online Tables A2 to A7 in the Appendix on the characteristics of included studies). Coders achieved 85% agreement on their codes and any discrepancies in coding were discussed and resolved. Self-esteem and self-compassion were most frequently assessed by the Rosenberg (1979) self-esteem scale and the Self-Compassion Scale (SCS; Neff, 2003), respectively. For self-concept and self-awareness, studies primarily used the Self Perception Profile for Children (SPPC; Harter, 1985), the Multidimensional Self-Concept Scale (Bracken, 1992), and the Self-Description Questionnaire (Marsh, 1990), and the Self-Consciousness Scale (Fenigstein et al., 1975). Self-agency was commonly assessed by the General Self-Efficacy Scale (Schwarzer & Jerusalem, 1995). For self-regulation, there was no predominant scale used. We combined self-esteem and self-concept (hereafter refers to as self-esteem/self-concept) because the latter is often operationalized as some form of evaluation of the self across different domains, such as physical appearance, social and academic competencies. Thus, we examined the relations of five self traits with depression and anxiety.

### Risk of bias (quality) assessment

Studies included in the meta-analysis were independently evaluated for quality by two reviewers using a modified Downs and Black (1998) instrument. The modified checklist utilizes 14 items to evaluate cross-sectional studies and 16 items for longitudinal studies, generating a total score ranging up to 14 or 16 points, respectively, with higher scores indicating greater quality. The instrument assessed five domains of study quality: external validity, study bias, confounding, selection bias, and study power, and include questions that require yes (1) or no (0) answers (e.g. ‘Is the hypothesis/aim/objective of the study clearly described?’). Most studies indicated moderate to high quality (*M* = 8.36, s.d. = 1.08) and had scores that ranged from 4.38 to 10.

### Multiple dependent effect sizes

There were several instances in which studies contributed multiple dependent effect sizes to our meta-analyses. Following the guidelines provided by Quintana (2015), we dealt with this issue in three ways. First, for cases examining more than one facet of selfhood with adolescent mental health, the different effect sizes were used to conduct the following analyses for depression and anxiety separately: (a) self-esteem/self-concept; (b) self-compassion; (c) self-awareness, (d) self-efficacy, and (e) self-regulation. Second, some studies measured each self traits in a variety of ways, for instance, self-esteem/self-concept was assessed by different measures, and there were also instances where studies included multiple indicators of depression and anxiety. For such cases, we computed the average effect size across all measures of the same facet of selfhood and all measures of the same mental health outcome within a study (Brewin, Kleiner, Vasterling, & Field, 2007), such that each study contributed only one effect size for the analyses involving each facet of selfhood with depression and anxiety.

Third, for longitudinal studies that provided correlations on the concurrent relations between selfhood and mental health outcomes (e.g. T_1_ selfhood with T_1_ depression/anxiety) and cross-lagged associations (e.g. T_1_ selfhood with T_2_ depression/anxiety, T_1_ depression/anxiety with T_2_ selfhood), there were multiple effect sizes. We computed the average effect sizes for each of these associations: (a) selfhood → mental health (e.g. T_1_ selfhood with T_2_ depression/anxiety, T_2_ selfhood with T_3_ depression/anxiety), (b) mental health → selfhood (e.g. T_1_ depression/anxiety with T_2_ selfhood, T_2_ depression/anxiety with T_3_ selfhood), and (c) selfhood ←→ mental health (e.g. T_1_ selfhood with T_1_ depression/anxiety, T_2_ selfhood with T_2_ depression/anxiety). In cases where more than one set of data was collected from the same study, we need to consider issues of statistical dependency that stem from the multiple dependent effect sizes (Hunter & Schmidt, 2004). We used the robust variance estimation to account for non-independent effect sizes, considering that it can also be adjusted to deal with smaller meta-analyses (*n* < 40; Fisher & Tipton, 2015).

### Computation of effect sizes

Pearson's correlation coefficients were used as the effect sizes, but these values first were converted into Fisher's z scale because they are not normally distributed. All effect sizes were transformed back into correlation coefficients for reporting the average effect sizes for the associations of selfhood with adolescents' depression and anxiety. We included 5 forest plots to visualize the effect sizes and confidence intervals (CIs) for five self traits, with a computed summary effect size for each plot. All analyses were conducted using R studio with the ‘metafor’ and ‘robumta’ packages (Fisher & Tipton, 2015; R Development Core Team, 2015; Viechtbauer, 2010). With high heterogeneity among studies included in the meta-analyses (see Table 2; Higgins, Thompson, Deeks, & Altman, 2003), we adopted the random-effect model for the meta-analyses, as well as moderator analyses. This model has the assumption of study heterogeneity – that is, the amount of variation in effect sizes that is derived from both study error and true study heterogeneity (study populations can vary in procedures, measures and settings).

**Table 2.** The effects of five facets of selfhood on adolescents' mental health

Selfhood	Depression/Anxiety					
	*k*	N*	*r*	95% CI	*Q*	*I*^2^
Self-esteem & self-concept	278/105	203 916/75 595	−0.52**/–0.36**	(−0.57 to −0.45)/(−0.40 to −0.32)		
Self-compassion	31/32	21 101/11 524	−0.45**/–0.43**	(−0.49 to −0.40)/(−0.48 to −0.38)	14 909.76/6421.16	97.80/97.17
Self-awareness	35/16	25 348/4096	−0.41**/–0.35**	(−0.47 to −0.35)/(−0.43 to −0.27)		
Self-efficacy	26/9	17 695/14 265	−0.36**/–0.36**	(−0.42 to −0.29)/(−0.44 to −0.27)		
Self-regulation	19/7	5666/4256	−0.30**/–0.31**	(−0.36 to −0.24)/(−0.44 to −0.17)		

*k*, number of effect sizes; N, sample size; *r*, effect sizes; C. I., confidence interval; *Q*, the ratio of observed variance to within-study variance; *I*^2^, percentage of observed variation that can be attributed to the actual differences between studies, rather than within-study variance.

**p < 0.0001.

To ascertain true heterogeneity in effect sizes, we used the Q-statistic, which calculates the ratio of observed variance to within-study variance. This statistic indicates how much of the overall heterogeneity can be attributed to true between-studies variation. A significant Q-statistic provides evidence that the included studies do not share a common effect size. However, a caveat about Q-statistic is that it underestimates heterogeneity in small samples and overestimates that in large samples. Thus, as Higgins et al. (2003) recommend, we included the *I^2^* statistic – a percentage that indicates the proportion of observed variation that is attributed to the actual differences between studies, rather than within-study variance. *I^2^* of 25, 50, and 75% represent low, moderate, and high variance, respectively. Compared to the Q-statistic, *I^2^* is not sensitive to the number of studies included and allows for CIs to be calculated (Higgins et al., 2003).

### Outline of steps in the analyses

We conducted two sets of meta-analyses on the association between the five self traits and two mental health outcomes – depression and anxiety. We further conducted moderation analyses using meta-regressions with the five self traits as a moderator to determine if the effect sizes of the five traits' associations with each mental health outcome differed. We conducted meta-regressions with individual factors (age and type of informants) and causal directions as moderators of the associations between selfhood and mental health. Last, publication bias was tested.

## Results

### Sample characteristics

Summary statistics of included articles' sample characteristics are presented in online Tables A2 to A7 in the Appendix. Of 558 effect sizes included in the meta-analyses, there were 383 independent effect sizes involving 192 248 adolescents for the link of self-esteem/self-concept with depression and anxiety, 63 effect sizes with 19 686 adolescents for the link of self-compassion to depression and anxiety, and 51 effect sizes with 35 204 adolescents on self-awareness and the link to the two mental health outcomes. The remaining 35 and 26 effect sizes were on self-efficacy and self-regulation, involving 20 564 and 6668 adolescents, respectively. Most of the studies were cross-sectional in design (75.5%; *n* = 224); 23.5% were longitudinal (*n* = 69) and 1% were RCTs (*n* = 3). These data were obtained from studies conducted across 39 countries (including UK, USA, Australia, Belgium, Canada, China, Costa Rica, Egypt, Hong Kong, India, Indonesia and Vietnam). Most of the study participants were from the United States of America (43.2%; *n* = 127) or countries in Europe (29.6%; *n* = 87). The average age of the sample ranged from 10 to 22 and the female proportion ranged from 31.1% to 94.5%.

**Figure 2.**
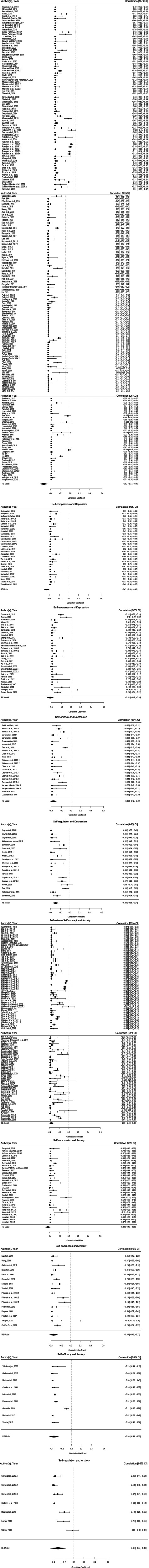
Forest plot of data investigating the relationship between adolescents' selfhood and mental health.

For each of the mental health outcomes – depression and anxiety, we presented 5 forest plots to visualize the effect sizes and CIs from the included studies, with computed summary effect sizes. In each of the 5 plots, each study is represented by a point estimate and is bounded by a CI for the effect. At the bottom of the plot, the summary effect size is represented by the polygon, with its width representing 95% CI. Studies with larger squares have contributed more to the summary effect size compared to other studies (refer to Fig. 2).

### How does selfhood relate to adolescent depression and anxiety?

For depression, self-esteem/self-concept had a high negative association, *r* = −0.52, *p* < 0.001; (95% CI −0.57 to −0.45). Self-compassion, *r* = −0.45, *p* < 0.001; (95% CI −0.49 to −0.40), self-awareness, *r* = −0.41, *p* < 0.001; (95% CI −0.47 to −0.35), and self-efficacy, *r* = −0.36, *p* < 0.001; (95% CI −0.42 to −0.29), had moderate negative associations. Self-regulation was also negatively related to depression, though the relationship was weaker, *r* = −0.30, *p* < 0.001; (95% CI −0.36 to −0.24). *Q* statistics analyses comparing the relations of the five self traits and depression indicated that their effect sizes differed significantly (*Q_between_* = 24.02, *p* = 0.0002). Self-esteem/self-concept and self-compassion had similar associations with depression – their effect sizes were not significantly different, and these associations were significantly greater than those involving self-awareness, self-efficacy, and self-regulation (refer to Table 2).

Self-esteem/self-concept, *r* = −0.36, *p* < 0.0001; (95% CI −0.40 to −0.32), self-compassion, *r* = −0.43, *p* < 0.0001; (95% CI −0.48 to −0.38), self-awareness, *r* = −0.35, *p* < 0.0001; (95% CI −0.43 to −0.27), self-efficacy, *r* = −0.36, *p* < 0.0001; (95% CI −0.44 to −0.27), and self-regulation, *r* = −0.31, *p* < 0.0001; (95% CI −0.44 to −0.17), had moderate negative associations with anxiety. None of these associations differed significantly from each other (*Q_between_* = 2.582, *p* = 0.630; refer to Table 2). Taken together, these findings demonstrated that greater self-esteem/self-concept, self-compassion, self-awareness, self-efficacy, and self-regulation are related to better mental health among young people, as indexed by their experience of lower depression and anxiety. The effect sizes were also far from trivial, ranging from 0.28 to 0.52, with most around 0.40. In general, the effect sizes did not differ from each other, with two main exceptions: the links for self-esteem/self-concept and self-compassion with depression were stronger than the others.

### What individual factors modulate the selfhood links with depression and anxiety?

#### Age

Average age of the sample was used as a continuous predictor in testing its moderating effects on the relationships between the five traits of self-hood with depression and anxiety. We found that age moderated the association between young people's self-esteem/self-concept and depression (*Q_between_* = 6.12, *p* = 0.013), with larger effect sizes in samples containing older adolescents (refer to Table 3). Put differently, older youths' self-esteem/self-concept was more strongly related to their lower experience of depression than their younger counterparts. Anxiety was unrelated to age and gender of sample.

**Table 3.** Moderators of the effects of adolescent selfhood and mental

Moderator	Depression					Anxiety				
	*k*	*r*	95% CI	Between group *Q*	*I*^2^	*k*	*r*	95% CI	Between group *Q*	*I*^2^
Age										
Self-esteem/self-concept	**177**	**0.26**	**(0.00–0.48)**	6.12*	**98.05**	77	0.26	(−0.07 to 0.54)	1.13	97.29
Self-compassion	29	−0.01	(−0.03 to 0.02)	0.54	90.89	23	0.26	(−0.28 to 0.73)	0.47	92.62
Self-awareness	27	0.01	(−0.03 to 0.05)	0.21	95.46	10	0.88	(0.03–1.70)	1.50	93.55
Self-efficacy	16	−0.02	(−0.06 to 0.02)	0.92	94.68	6	−0.56	(−2.72 to 1.45)	0.66	98.09
Self-regulation	14	−0.00	(−0.02 to 0.01)	0.31	76.42	4	−0.14	(−1.46 to 1.05)	0.29	90.13
Types of informants										
Self-esteem/self-concept										
Adolescents		0.53	(0.49–0.56)				**0.44**	**(0.40–0.48)**		
Parents	214	0.40	(0.23–0.57)	3.67	98.09	**88**	**0.14**	**(−0.07 to 0.34)**	14.72***	**96.77**
Mixed (Adolescents and Parents)		0.46	(0.25–0.77)				**0.16**	**(−0.05 to 0.55)**		
Self-compassion										
Adolescents	–	–	–	–		–	–	–	–	
Parents		–	–	–	–	–	–	–	–	–
Mixed (Adolescents and Parents)		–	–	–		–	–	–	–	
Self-awareness										
Adolescents		0.43	(0.36–0.50)				–	–		
Parents	31	0.31	(0.21–0.77)	0.38	96.17	–	–	–	–	–
Mixed (Adolescents and Parents)		–	–				–	–		
Self-efficacy										
Adolescents		**0.40**	**(0.33–0.47)**				–	–		
Parents	**19**	**–**	**–**	4.24*	**90.53**	–	–	–	–	–
Mixed (Adolescents and Parents)		**0.00**	**(0.02–0.77)**				–	–		
Self-regulation										
Adolescents		**0.29**	**(0.24–0.35)**				–		–	
Parents	**14**	**–**	**–**	10.02**	**66.47**	–	–	–		–
Mixed (Adolescents and Parents)		**0.08**	**(−0.10 to 0.26)**				–			
Causal direction										
Self-esteem/self-concept										
Selfhood→Mental Health		**−0.54**	**(−0.57 to 0.51)**				−0.43	(−0.47 to 0.38)		
Well-being→Mental Health	**268**	**−0.39**	**(−0.50 to 0.28)**	20.27***	**98.09**	98	−0.34	(−0.39 to 0.14)	2.86	97.35
Selfhood←→Mental Health		**−0.40**	**(−0.40 to 0.29)**				−0.32	(−0.49 to 0.14)		
Self-compassion										
Selfhood→Mental Health	–	–	–	–	–	31	−0.45	(−0.53 to 0.39)	0.52	93.12
Well-being→Mental Health		–	–				−0.50	(−0.92 to 0.09)		
Selfhood←→Mental Health		–	–				−0.54	(−0.88 to 0.20)		
Self-awareness										
Selfhood→Mental Health		**−0.44**	**(−0.51 to 0.39)**				−0.36	(−0.45 to 0.26)		
Well-being→Mental Health	**34**	**−0.19**	**(−0.48 to 0.11)**	7.14*	**95.30**	13	−0.11	(−0.31 to 0.33)	3.26	91.50
Selfhood←→Mental Health		**−0.12**	**(−0.55 to 0.38)**				−0.49	(−0.84 to 0.16)		
Self-efficacy										
Selfhood→Mental Health		**−0.40**	**(−0.48 to 0.33)**				–	–		
Well-being→Mental Health	**23**	–	–	4.58*	**92.41**	–	–	–	–	–
Selfhood←→Mental Health		**−0.19**	**(−0.46 to 0.08)**				–	–		
Self-regulation										
Selfhood→Mental Health		−0.31	(−0.39 to 0.23)				−0.30	(−0.52 to 0.08)		
Well-being→Mental Health	18	−0.32	(−0.63 to 0.03)	0.81	88.09	7	−0.26	(−0.42 to 0.07)	0.212	94.27
Selfhood←→Mental Health		−0.23	(−0.29 to 0.04)				−0.17	(−0.32 to 0.16)		

*k*, number of effect sizes; N, sample size; *r,* effect sizes; C. I., confidence interval; *Q*, the ratio of observed variation to within-study variance; *I*^2^, percentage of observed variation that can be attributed to the actual differences between studies, rather than within-study variance. We did not include moderator effect if there are no studies examined those factors in the selfhood-mental health links. Moderator effects in bold are significant.

*p < 0.05; **p < 0.01; ***p < 0.001.

#### Type of informants

Type of informant moderated the relations of young people's self-efficacy with depression (*Q_between_* = 4.24, *p* = 0.039), self-regulation with depression (*Q_between_* = 10.02, *p* = 0.0015), and self-esteem/self-concept with anxiety (*Q_between_* = 14.72, *p* = 0.0006). In particular, the associations were greater when youths were the reporters than when parents were the informants or when both youths and parents were the reporters (for different measures on selfhood, depression and anxiety). In general, these findings suggest that stronger relationships are found in youth's own self-reports than when ratings are obtained from their parents.

### What is the causal direction of adolescent selfhood with depression and anxiety?

Using longitudinal studies, we illuminated the causal connections between youth selfhood and depression and anxiety by comparing the following directionality of influence: (a) selfhood ←→ mental well-being, (b) selfhood → mental well-being, and (c) mental well-being→ selfhood. Directionality of influence significantly moderated the links of self-esteem/self-concept (*Q_between_* = 20.27, *p* < 0.0001), self-awareness (*Q_between_* = 7.14, *p* = 0.0282), and self-efficacy with depression (*Q_between_* = 4.58, *p* = 0.032, respectively). The bidirectional negative causation between self-esteem/self-concept and depression was significantly greater than depression leading to low self-esteem/self-concept and low self-esteem/self-concept driving depression. The bidirectional causation between self-awareness and depression was significantly greater than low self-awareness leading to depression, but not significantly greater than depression predicting low self-awareness. Similarly, the bidirectional causation between self-efficacy and depression was greater than low self-efficacy driving depression (there are no studies on depression driving self-efficacy). Directionality of influence did not moderate the self-regulation and depression association (*Q_between_* = 0.81, *p* = 0.667) and the associations of self-esteem/self-concept (*Q_between_* = 2.86, *p* = 0.239), self-compassion (*Q_between_* = 0.52, *p* = 0.77), self-awareness (*Q_between_* = 3.26, *p* = 0.195), and self-regulation (*Q_between_* = 0.212, *p* = 0.899) with anxiety. For self-compassion and depression, as well as for self-efficacy and anxiety, studies included in the meta-regression examined concurrent relations and directionality of influence was not investigated as a moderator (refer to Table 3).

### Publication bias analysis

**Figure 3.**
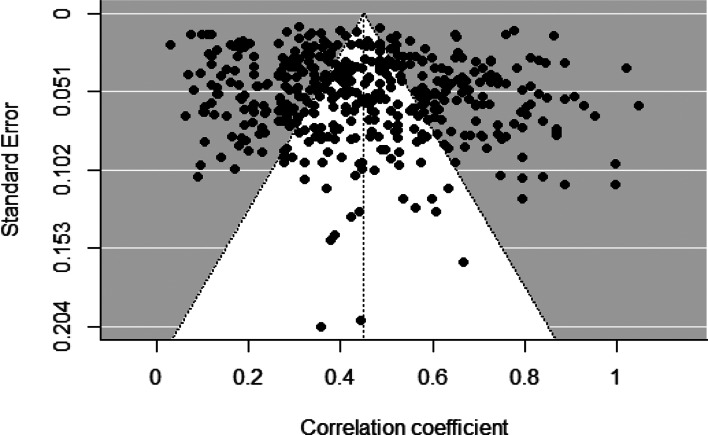
Funnel plot of the effect sizes of adolescents' selfhood with mental health. *Note.* The funnel lines indicate the degree of spread that is expected for a given level of standard error and are centered on the summary effect size that is represented by the vertical line. Data points scattered symmetrically on both sides of the funnel line and has the shape of an even funnel provide evidence for an unbiased sample.

Three analyses were used to ascertain publication bias. First, funnel plots can reveal possible publication bias, in which a cluster of data is non-symmetrical and deviates from the shape of a funnel. Figure 3 provides the funnel plot analyses for our study. The points that fell into the gray area were asymmetrical and indicated that publication bias may exist. Because asymmetry may reflect other types of bias, such as study quality (Egger, Davey Smith, Schneider, & Minder, 1997), funnel plots offer only a subjective measure of potential publication bias. Two other objective measures of potential bias – the rank correlation test and Egger's regression test (Begg & Mazumdar, 1994; Sterne, Gavaghan, & Egger, 2000) – yielded non-significant results, which indicate an absence of publication bias for studies included in our meta-analyses. Taken together, these three analyses suggested that publication bias has not seriously distorted our findings.

## Discussion

Our meta-analysis found abundant evidence that selfhood development relates to adolescent mental health. Our analysis involving 558 effect sizes from 298 studies found that various facets of selfhood and their associated traits had moderate negative associations with depression and anxiety – two most prevalent mental health conditions that affect adolescents worldwide (Eisenberg et al., 2007; Young, Klap, Shoai, & Wells, 2008). Young people with greater self-esteem/self-concept, self-compassion, self-awareness, self-efficacy and self-regulation experienced lower depression and anxiety. The strongest relationships to depression were found with self-esteem/self-concept and self-compassion. With anxiety, all five self traits had roughly similar relationships. These findings suggest how different self traits factor in specific form of psychopathology among adolescents (Baumeister, 2022; Luyten & Fonagy, 2016). Our meta-analytic review highlights the need to investigate multiple self-variables conjointly to understand their differential relations to specific mental health conditions among adolescents.

## Key findings

Our findings indicate that individuals who build a well-organized knowledge structure about their competencies, values, and memories, and establish greater consciousness of what the self is like (both privately and publicly) experience less depressive and anxiety symptoms. Belief in one's competency to effect change, overcome difficulties, and persist in the face of setbacks, and the ability to regulate emotions, behaviors, and cognitions relate to positive mental health functioning. Our meta-regressions comparing the associations of different self traits with adolescent depression and anxiety provide insights into a nuanced understanding of self traits that are pivotal in contributing to mental health functioning, especially depression. Adolescents are more sensitive to social cues in evaluating the self (Larson, Moneta, Richards, & Wilson, 2002), and they often engage in social comparisons that can result in value judgment about the self (Ke, Wu, Willner, Brown, & Crowley, 2018). These developments underscore the role of self-esteem/self-concept and self-compassion in depression among adolescents (Liu et al., 2021; Tynes, Umaña-Taylor, Rose, Lin, & Anderson, 2012).

Our meta-analysis found that researchers have focused disproportionately on the mental health correlates of self-esteem/self-concept, and less on self-compassion, self-awareness, self-efficacy and self-regulation (e.g. Prinstein & La Greca, 2002; Watson, 2006). Most (68.6%) of the effect sizes we found were directed at the connections between self-esteem/self-concept and youth depression and anxiety. Our meta-analytic findings, however, challenge the wisdom of over-emphasizing this association and highlight the need to focus also on self-compassion and self-awareness. According to self-theories of depression (Luyten & Fonagy, 2016), an accurate mental representation of the self, and the ability to engage in introspection without overly critical self-judgment provide the basis for a stable and coherent sense of self that guards against social dependency and excessive self-criticism – common experience of depressed individuals (Luyten & Fonagy, 2016). At the same time, it may be prudent for researchers to turn more attention to the role of self-efficacy and self-regulation among young people. We found these self traits were relatively infrequently studied (accounting for approximately 10.9% of effect sizes) in terms of their relationships with depression and anxiety. Our results revealed that to a modest but significant degree, adolescents' self-efficacy and self-regulation are associated with lower symptoms of depression and anxiety. With growing autonomy and agency, adolescents develop greater self-efficacious beliefs and learn self-regulatory behaviors that have important implications for their positive mental health functioning (Rudolph, Lambert, Clark, & Kurlakowsky, 2001; Watters & Wojciak, 2020).

The links between self traits and mental health varied considerably. Some self traits, such as self-esteem/self-concept, relate similarly to both depression and anxiety. However, for specific form of psychopathology, certain self traits are more important than the others (Baumeister, 2022; Harter, 1993; Luyten & Fonagy, 2016). Our findings indicate that self-esteem/self-concept, self-awareness, and self-compassion may differ from self-efficacy and self-regulation in relating to depression among adolescents. In contrast, various self traits may have similar relations to and meanings for adolescents' anxiety. Existing studies with adolescents, however, rarely make reference to a model of selfhood and consider how different facets of the self and their associated traits relate to specific form of psychopathology. The few studies that examined conjointly different self traits found that they independently predicted adolescent mental health outcomes [e.g. self-compassion predicted depression after controlling for self-esteem (e.g. Barry et al., 2015; Stolow, 2016; Wasylkiw et al., 2012)], and the moderate associations among different self traits suggested that they are empirically related but distinct. Findings from these few studies and our meta-analytic review suggest that future research should measure multiple self traits to understand their differential relations with specific mental health conditions among adolescents.

Adolescent age moderated the strength of associations between self traits and mental health. In particular, older adolescents with higher self-esteem/self-concept experienced lower depression than their younger counterparts. Older adolescents' cognitive advancement (Steinberg, 2010), which facilitates reflection and formation of a positively biased perception of the self (Moneta, Schneider, & Csikszentmihalyi, 2001), helps them navigate life challenges and stressors (Sheldon, 2012) and manage the associated emotional lability – precursors to the development of depression (Maciejewski et al., 2014; Silk, Steinberg, & Morris, 2003). Source of information (parents, or the adolescents themselves) was also an important moderator. The associations of self-efficacy and self-regulation with depression, and self-esteem/self-concept with anxiety were greater for adolescents' reports than in parents' ratings. Adolescents' perception of these self traits factor more in their experience of psychopathology because of greater autonomy and decision-making (Zimmer-Gembeck & Collins, 2003), as well as increased engagement in social comparisons during this developmental phase (Ke et al., 2018). The absence of differences between parental and adolescent reports for the associations of other self traits with depression and anxiety (e.g. self-esteem/self-concept with depression) suggests that parents may seek sources of accurate information beyond the child, such as siblings, peers and teachers with whom the adolescents interact (Hope et al., 1999; Smith, 2007). These findings underscore the need for investigators to pay more attention to the content of selfhood information that adolescents and parents provide, and to include a broader frame of reference when investigating the associated mental health implications.

We compared three models with longitudinal studies to establish the likely causal sequence: self traits causing anxiety and depression, the reverse, or bidirectional. The main conclusion was that bidirectional influence was the best supported model for depression (Only self-regulation failed to show any difference among three causal models of depression). Our findings are consistent with theories and empirical work that elucidated the reciprocal interplay of selfhood and psychopathology (Beck, 1967; Teasdale & Fogarty, 1979). In particular, selfhood can lead to psychopathology because a coherent and stable sense of self allow individuals to introspect and establish a balanced cognitive state that is important for positive mental health functioning. Conversely, psychopathology can drive mental health state by affecting individuals' cognitive vulnerability, particularly heightened awareness of or access to negative cognitions that further perpetuate psychopathology. Our findings revealed that depression and self traits reciprocally influence each other, which is consistent with the centrality of the self and social comparison that can result in value judgment and negative cognitions during adolescence (Harter, 2012; Ke et al., 2018). Depression emerges interactively in step with low self-esteem, lack of self-compassion, low self-awareness, and low self-efficacy. For anxiety, there was about equal evidence for each of the three causal models. The links to anxiety remain less well understood based on current evidence. For example, although social anxiety is strongly correlated with low self-esteem (Baumeister, 2022), it is unclear what causes what. The ambiguity regarding anxiety may be partly due to the lesser amount of available evidence.

## Theoretical and practical implications

Recent efforts suggest that unpacking the conceptual model of selfhood has been fruitful, particularly in understanding the role of different facets of the self in the development of psychopathology (Baumeister, 2022). Most studies examining adolescent selfhood and mental health focused on the facet of self-knowledge and its associated traits (e.g. Charoensuk, 2007; Lee & Hankin, 2009). To build and expand the selfhood model, there is a need to shift towards more studies that investigate other facets of selfhood, and the associated traits, to understand the psychological mechanism involving the self and youth mental health. The few studies that have examined conjointly different self traits revealed differential linkages with youth depression and anxiety (e.g. Stolow, 2016; Wright, 2005). These findings underscore a nuanced approach in understanding how different facets of selfhood and their related traits account for specific form of psychopathology among adolescents.

Importantly, the different facets of selfhood and their associated traits may reflect varying amount of conceptual overlap with specific form of psychopathology, such as how low self-esteem is often implicated in negative cognition that is inherent to depression (e.g. Zimmerman & Coryell, 1994), which can result in overestimation of the associations of self traits and psychopathology (Kaplan, 2009). A step forward in aligning the conceptualization and operationalization of different self traits and specific form of psychopathology is to synthesize items from all measures and examine their underlying factor structures. Strong overlap in the conceptualization of self traits and specific mental health condition can be reflected in the correlation of two constructs that is above 0.90, which provides empirical evidence that the two constructs are similar and should be regarded as one (Kaplan, 2009).

As can be seen from our meta-analysis, the moderate magnitude of associations of the five self traits (self-esteem/self-concept, self-compassion, self-awareness, self-regulation and self-efficacy) with depression and anxiety suggest modest overlap in the items measuring the five traits and the two mental health conditions. To gain precision in our understanding of adolescent selfhood and mental health implications, future work needs to address important conceptual and measurement issues, particularly to elucidate whether the conceptualizations of facets of selfhood (and their associated traits) and specific form of psychopathology are accompanied by clear distinctions in their operationalizations. Greater efforts are needed in deriving more sophisticated measures that attend to the conceptual distinctions of facets of selfhood and different forms of psychopathology. Setting this approach as a standard way of assessing youth selfhood and mental health would facilitate a comprehensive and converged understanding of the distinct pattern of associations of selfhood with different mental health conditions, as well as moderators of these associations.

From a practical perspective, our findings indicate the desirability of paying attention to the advantages of teaching young people means to build self-knowledge, interpersonal self, and self-agency as psychological skills they can cultivate for mental well-being. There are practical payoffs in going beyond self-esteem to look more closely at the role of other self traits, such as self-compassion (Neff, 2011). Interventions and programs that target the intra- and interpersonal resources and competencies of young people to enhance mental health need to consider the different facets of selfhood and their dynamics. Advising schools and practitioners on how to design and implement evidence-based training programs for youth mental health that translate the conceptual model and empirical findings on selfhood should be particularly helpful. Likewise, helping parents understand their perspectives and those of their adolescent-children about selfhood development will enable parents to distinguish circumstances when their views differ from those of their adolescent-child, which is more pivotal in driving their own mental health, as documented in our meta-analysis.

## Limitations and conclusions

As with any meta-analyses, the conclusions we drew were constrained by the available studies. We intended to examine mental health broadly, but the available information mostly focused on anxiety and depression. To be sure, these are the two most prevalent mental health conditions that affect adolescents worldwide (Eisenberg et al., 2007; Wellcome Trust, 2021; Young et al., 2008), but there are many other important but less common and less well studied mental health problems. Future research may profit by examining whether and how other mental health problems relate to adolescent selfhood. The differences we found may reflect differential contributions of various self traits to mental health issues, but it is also possible that the quality of the measures was different for different traits, and so stronger results simply reflect more precise measurement. In particular, self-regulation was not measured in a consistent fashion, and research with adults has only recently benefited from improved trait measurement (e.g. Tangney, Baumeister, & Boone, 2004). We also caution against always using the same measure, and for advancing theory, future research may benefit from improved measurements and the employment of different measures.

Even in our sample, the amount of information was variable, thus calling for caution with regard to sweeping conclusions. Apart from the many studies involving self-esteem, the number of studies on the other self traits and youth psychopathology, especially anxiety, is relatively small. To provide a more robust understanding of the self in youth mental health, there is a need to unpack the tripartite mode of selfhood in understanding how different facets of the self and their associated traits relate to specific form of psychopathology among adolescents. Particularly, longitudinal and experiment investigations can illuminate the directions of causation and contribute to an evidence-based approach in building aspects of the self for youth mental health functioning.

## Data Availability

All data generated or analyzed during this study are included in this published article (and its supplementary information files).

## Supplementary material

For supplementary material accompanying this paper visit https://doi.org/10.1017/S0033291723001083. click here to view supplementary material
