# How Do Aspects of Selfhood Relate to Depression and Anxiety among Youth? A Meta-Analysis – CORRIGENDUM

**DOI:** 10.1017/S0033291723001691

**Published:** 2023-09

**Authors:** GeckHong Yeo, Cameron Tan, Dean Ho, Roy F. Baumeister

**Affiliations:** 1 N.1 Institute for Health, National University of Singapore, Singapore; 2 The Institute for Digital Medicine (WisDM), Yong Loo Lin School of Medicine, National University of Singapore, Singapore; 3 Department of Biomedical Engineering, NUS Engineering, National University of Singapore, Singapore; 4 Department of Pharmacology, Yong Loo Lin School of Medicine, National University of Singapore, Singapore; 5 School of Psychology, University of Queensland, Brisbane, QLD, Australia

When this article was originally published in Psychological Medicine it omitted an acknowledgment credit. This section has been updated to include the following: ‘The authors also grateful acknowledge research assistant Qiao Ying Leong and research fellow Dr. Alexandria Marie Remus of the N.1 Institute for Health and WisDM for their work in Prospero registration/literature review framework guidance, and literature review.’.

The authors apologise for this error.
