# Supplementary material for: How do aspects of selfhood relate to depression and anxiety among youth? A meta-analysis
Source: Psychol Med. 2023 May 22;53(11):4833–55. doi: 10.1017/S0033291723001083 (PMC10476091; doi:10.1017/S0033291723001083)
Supplement: Supplementary file 1 [file S0033291723001083sup001.docx]

**Appendix**

Table A1

*Search Terms for Search String Development using the PICO Concept*

| Database | Search String | Number of outcomes |
| --- | --- | --- |
| Pubmed | (Adolescent[Title/Abstract] OR Young people[Title/Abstract] OR young adult[Title/Abstract] OR youth[Title/Abstract] OR adolescent[Title/Abstract] OR youth[Title/Abstract])  AND (Anxiety[MeSH] OR Depression[MeSH] OR loneliness[MeSH] OR well-being[MESH] OR distress[MESH] OR internalizing symptoms[MESH] OR externalizing symptoms[MESH])  And (self-esteem[MESH] OR self-worth[MESH] OR self-regard[MESH] OR self-evaluation[MESH] OR  self-compassion[MESH], self-blame[MESH]; self-acceptance[MESH] OR self-concept[MESH] OR self-understanding[MESH] OR self-description[MESH] OR self-identity[MESH] OR self-awareness[MESH] OR self-image[MESH] OR self-liking[MESH] OR self-confidence[MESH] OR self criticism[MESH] OR self-efficacy[MESH] OR  self-regulation[MESH] OR emotional regulation[MESH] OR self-control[MESH] OR self-autonomy[MESH] OR self determination[MESH] OR self-consciousness[MESH] OR self-competence[MESH] OR self-presentation[MESH]) | 27588 |
| Embase | ((adolescents OR teenagers OR young) AND adults OR teen OR youth)  AND (Anxiety OR Depressio OR lonelinesS OR well-being OR distress OR internalizing symptom OR externalizing symptoms)  AND (self-esteem OR self-worth OR self-regard OR self-evaluation OR self-compassion, self-blame; self-acceptance OR self-concept OR self-understanding OR self-description OR self-identity OR self-awareness OR self-image OR self-liking OR self-confidence OR self criticism OR self-efficacy OR  self-regulation OR emotional regulation OR self-control OR self-autonomy OR self determination OR self-consciousness OR self-competence OR self-presentation)) | 6806 |
| CINAHL | TI (adolescents or teenagers or young adults or teen or youth) OR AB (adolescents or teenagers or young adults or teen or youth)  AND anxiety OR depression OR Mental Well-being (anxiety or depression) OR Mental Well-being (loneliness) OR Psychological Well-being (anxiety or depression) OR Psychological Well-being (loneliness) OR TI internalizing symptoms OR externalizing symptoms OR TX distress  AND TX (self-esteem or self-worth or self-regard or self-evaluation or self-compassion or self-blame or self-acceptance or self-concept or self-understanding or self-description or self-identity or self-awareness or self-image or self-liking or self-confidence or self criticism) OR TX (self-efficacy or self-regulation or emotional regulation or self-control or self-autonomy or self determination or self-competence) OR TX (self-consciousness or self-presentation) | 7850 |
| PsyInfo | (adolescents or teenagers or young adults or teen or youth).ti. or (adolescents or teenagers or young adults or teen or youth).ab.  and (anxiety or depression or Mental Well-being or loneliness or Psychological Well-being or internalizing symptoms or externalizing symptoms or distress).af.  and (self-esteem or self-worth or self-regard or self-evaluation or self-compassion or self-blame or self-acceptance or self-concept or self-understanding or self-description or self-identity or self-awareness or self-image or self-liking or self-confidence or self criticism).ab. or (self-efficacy or self-regulation or emotional regulation or self-control or self-autonomy or self determination or self-competence).ab. or (self-consciousness or self-presentation)ti. | 29370 |
| Total | | 71614 |

**Table A2**

*Characteristics of Studies on Adolescent Self-Esteem and Mental Health Included in the Meta-Analyses*

| Author(s) | Year | N | *r* | M_age_ (age range) | Female | Culture | Design | Outcomes |
| --- | --- | --- | --- | --- | --- | --- | --- | --- |
| ^a^Adams | 2008 | 276 | 0.37 | 13.07 (11-15) | 57.97 | W | L | D |
| ^a^Albert | 2003 | 197 | 0.65 | - (-) | 61 | W | C | D |
| ^a^Armstrong et al. | 2015 | 813 | 0.73 | 16.15 (13-19) | 52 | W | C | D |
| ^a^Auerbach et al. | 2010 | 160 | 0.62 | 15.17 (12-18) | 54 | W | L | D |
| ^a^Babore et al. | 2016 | 594 | 0.58 | 12.12 (10-14) | 50 | W | C | D |
| ^a^Bang et al. | 2020 | 273 | 0.67 | - (11-18) | 53.1 | W | L | D |
| ^b^Bardone-Cone et al. | 2013 | 441 | (0.52) | 18.71 (17-24) | 100 | W | C | A |
| ^a^Barry et al. | 2015 | 251 | 0.27 (0.32) | 16.78 (16-18) | - | W | C | AD |
| ^a^Barutçu Yıldırım and Demir | 2020 | 801 | (0.42) | - (18-25) | 50.4 | E | C | A |
| ^a^Bohon et al. | 2003 | 496 | 0.26 | 16.5 (15-18) | 100 | W | L | D |
| ^a^Bos et al. | 2010 | 264 | 0.41 (0.44) | 13.92 (-) | 57.57 | W | C | AD |
| ^a^Bosacki et al. | 2007 | 7290 | 0.55 (0.27) | 15.58 (13-18) | 51.52 | W | C | AD |
| ^b^Bowles | 2017 | 208 | (0.27) | 14.53 (11-17) | - | W | C | A |
| ^a^Brausch and Decker | 2014 | 392 | 0.69 | 15.04 (-) | 48.1 | W | C | D |
| ^b^Burwell and Shirk | 2006 | 110 | 0.35 | 13.62 (12-15) | 58.2 | W | L | D |
| ^a^Carter et al. | 2020 | 559 | 0.51 | 14.99 (13-17) | - | W | C | D |
| ^a^Cassidy et al. | 2004 | 154 | 0.48 (0.48) | 17.5 (14-21) | 55 | W | C | AD |
| ^a^Chang | 2001 | 268 | 0.57 | 15.77 (14-19) | 61.12 | W | C | D |
| ^a^Chapman | 2003 | 2099 | 0.37 | - (11-17) | 58 | W | C | D |
| ^a^Chapman | 2016 | 406 | 0.08 | 16.33 (17-19) | 56.2 | W | C | D |
| ^a^Charoensuk | 2007 | 812 | 0.38 | 16.2 (-) | 62 | E | C | D |
| ^b^Chavez | 2004 | 65 | 0.51 | 15.66 (13-18) | 53.8 | W | C | D |
| ^a^Chen and Qin | 2019 | 569 | (0.35) | 11.68 (10-15) | 49.4 | E | C | A |
| Author(s) | Year | N | *r* | M_age_ (age range) | Female | Culture | Design | Outcomes |
| ^b^Cheung | 2006 | 1043 | 0.47 | 16.04 (-) | 38.7 | E | C | D |
| ^b^Ching et al. | 2021 | 439 | 0.33 | 14.2 (13.9-14.6) | 100 | E | L | D |
| ^b^Choi and Choi | 2016 | 4995 | 0.45 | 15.99 (15-17) | 49.6 | E, W | C | D |
| ^b^Choi et al. | 2010 | 454 | 0.34 | 16.12 (14-18) | - | E | C | D |
| ^a^Cikrikci et al. | 2018 | 397 | (0.34) | 16.09 (14-19) | 50.6 | E | C | A |
| ^a^Civitci | 2010 | 255 | 0.44 | 12.92 (11-15) | 45.88 | E | C | D |
| ^a^Cong et al. | 2019 | 852 | 0.49 | 14.8 (-) | 49.4 | E | C | D |
| ^b^Crocker et al. | 2006 | 501 | (0.60) | - (14-15) | 100 | W | L | A |
| ^a^Dan et al. | 2014 | 327 | (0.44) | 16.4 (15-17) | 69.41 | E | C | A |
| ^b^Davis et al. | 2006 | 160 | 0.36 | 15.4 (-) | 47.5 | W | C | D |
| ^a^de Jong et al. | 2012 | 1806 | 0.65 (0.69) | 13.6 (-) | 54.98 | W | C | AD |
| ^a^Deepthi et al. | 2014 | 78 | 0.08 | - (12-17) | 48.7 | E | C | D |
| ^a^Dittrick | 2019 | 1001 | (0.39) | 13.62 (10-17) | 48.8 | W | C | A |
| ^b^Dou et al. | 2016 | 1108 | 0.45 | 14.65 (11-17) | 50.63 | E | C | D |
| ^a^Du et al. | 2015 | 384 | 0.45 | 14 (12-18) | 48.42 | E | C | D |
| ^a^Duru et al. | 2019 | 2018 | 0.36 (0.33) | 16.13 (-) | 54.36 | E | C | AD |
| ^b^Ellis | 2000 | 117 | 0.14 | 15.7 (-) | 100 | W | C | D |
| ^a^Fanti et al. | 2013 | 2306 | (0.35) | 16 (15-18) | 49.7 | E | L | A |
| ^b^Finkenauer et al. | 2005 | 1359 | 0.48 | 12.3 (10-14) | 47.8 | W | C | D |
| ^a^Fitzpatrick | 2009 | 227 | 0.74 | 16.3 (15-17) | 48 | W | C | D |
| ^b^Flett et al. | 2006 | 1250 | 0.51 | 17.66 (-) | 100 | W | C | D |
| ^a^Flett et al. | 2016 | 218 | 0.44 | 12.19 (-) | 50 | E | C | D |
| ^b^Fredriksen et al. | 2004 | 2,259 | 0.57 | 12 (11-14) | 49.6 | W | L | D |
| ^a^Gamble et al | 2005 | 134 | 0.61 | 16.2 (-) | 57.5 | W | C | D |
| ^a^Garaigordobil | 2015 | 286 | 0.21 | 14.72 (14-16) | 48.3 | W | C | D |
| ^a^Gardner et al. | 2019 | 334 | 0.70 (0.47) | 14.56 (10-18) | 49 | E | C | AD |
| ^a^Gardner et al. | 2019 | 334 | 0.60 | 14.74 (10-18) | 49 | E | C | D |
| Author(s) | Year | N | *r* | M_age_ (age range) | Female | Culture | Design | Outcomes |
| ^a^Garthe et al. | 2012 | 63 | 0.54 | 20.36 (17-25) | 73.02 | W | L | D |
| ^b^Gaylord-Harden et al. | 2007 | 227 | 0.29 (0.26) | 12.61 (-) | 63 | W | L | AD |
| ^a^George | 2014 | 477 | 0.64 | - (-) | - | W | L | D |
| ^a^Gerard et al. | 2004 | 5070 | 0.45 | - (11-18) | 49.3 | W | L | D |
| ^a^Gittins et al. | 2020 | 243 | 0.74 | 12.08 (11-13) | 52 | W | L | D |
| ^b^Goldstein | 2016 | 1464 | (0.38) | 12.84 (11-15) | - | W | L | A |
| ^a^Green et al. | 2018 | 724 | 0.69 | 13.83 (12-17) | 54 | W | L | D |
| ^b^Grills et al. | 2002 | 279 | (0.23) | 11.75 (11-13) | 53 | W | C | A |
| ^b^Grøholt | 2005 | 65 | 0.23 | 17 (13-19) | 90 | W | C | D |
| ^a^Guo et al. | 2018 | 847 | (0.32) | 12.96 (10-15) | 51.59 | E | L | A |
| ^a^Hamon et al. | 2012 | 213 | 0.55 | 19.34 (-) | 56.3 | W | C | D |
| ^a^Han et al. | 2017 | 788 | 0.45 | 19.6 (17-23) | 48.1 | E | C | D |
| ^a^Harris | 2006 | 90 | 0.06 | 16.3 (13-18) | 100 | W | C | D |
| ^a^Hatchel et al. | 2018 | 263 | (0.51) | 20.58 (-) | 50 | W | C | A |
| ^a^Hernández | 2006 | 225 | 0.48 | - (18-25) | 100 | W | C | D |
| ^a^Houtberg et al. | 2018 | 99 | 0.79 (0.40) | 22 (-) | 47.8 | W | C | AD |
| ^a^Hu et al. | 2014 | 370 | 0.68 | 15.12 (14-16) | 50.81 | E | C | D |
| ^a^Huang et al. | 2019 | 390 | 0.56 | 16.57 (15-19) | 42.6 | E | C | D |
| ^a^Hunter et al. | 2014 | 933 | 0.48 | - (10-14) | 50 | W | C | D |
| ^b^Hurd et al. | 2018 | 340 | 0.64 (0.39) | 18.11 (-) | 69 | W | L | AD |
| ^a^Huynh et al. | 2010 | 601 | 0.52 | 17.81 (17-18) | 51.2 | W | C | D |
| ^b^Ishizu | 2017 | 371 | 0.17 | 12.79 (12-14) | 50.4 | E | L | D |
| ^a^Issacs et al. | 2008 | 177 | 0.68 | - (14-15) | 57.1 | W | L | D |
| ^b^Ju and Lee | 2018 | 2844 | 0.45 | - (10-14) | - | E | L | D |
| ^a^Kam et al. | 2013 | 338 | 0.18 | 12.27 (11-14) | 100 | W | C | D |
| ^a^Kavehfarsani et al. | 2020 | 173 | 0.57 | - (12-16) | 100 | E | C | D |
| ^a^Kenny et al. | 2016 | 8121 | 0.65 | 20.42 (17-25) | 66 | W | C | D |
| ^a^Kiang et al. | 2020 | 175 | 0.37 | 12.86 (10-15) | 51.4 | W | C | D |
| Author(s) | Year | N | *r* | M_age_ (age range) | Female | Culture | Design | Outcomes |
| ^b^Kingery | 2011 | 365 | 0.58 | 11.17 (11-12) | 52.1 | W | L | D |
| ^a^Krishnan | 2004 | 208 | 0.27 | 15.26 (14-16) | 51.9 | E | C | D |
| ^a^Lan et al. | 2019 | 1210 | 0.35 | 13.63 (11-19) | 49.3 | E | C | D |
| ^a^Lee | 2016 | 1379 | 0.48 | 16.5 (12-18) | 51 | W | L | D |
| ^b^Lee et al. | 2014 | 350 | 0.60 (0.53) | 14.39 (-) | 57 | W | L | AD |
| ^a^Lee et al. | 2018 | 481 | 0.43 | 14 (13-15) | - | E | C | D |
| ^b^Lessard et al. | 2018 | 5991 | 0.36 (0.32) | - (11-13) | 52 | W | C | AD |
| ^a^Li and Patterson | 2016 | 232 | 0.13 | 20.75 (19-22) | 55.6 | E | C | D |
| ^a^Li et al. | 2010 | 1883 | 0.53 | 14.9 (12-19) | 48.9 | E | C | D |
| ^a^Li et al. | 2015 | 1045 | 0.55 | 14.35 (12-16) | 52.9 | E | C | D |
| ^a^Lin | 2015 | 814 | 0.61 | 20.13 (-) | 68.2 | E | C | D |
| ^a^Lin | 2019 | 2690 | 0.20 | - (13-17) | - | E | L | D |
| ^a^Litwack et al. | 2010 | 281 | 0.73 | 13.95 (13-15) | 58.01 | W | C | D |
| ^a^Lo Cascio et al. | 2013 | 350 | (0.72) | 14.9 (13-16) | 42.57 | W | C | A |
| ^a^Longmore | 2004 | 7965 | 0.38 | 15.1 (-) | 46 | W | L | D |
| ^a^Marcotte et al. | 2002 | 547 | 0.38 | 14.46 (11-18) | 51 | W | C | D |
| ^b^Margolin | 2006 | 161 | 0.56 (0.06) | - (11-12) | 62 | W | C | AD |
| ^a^Marshall | 2001 | 642 | 0.35 | 17.3 (-) | 51.4 | W | C | D |
| ^b^Massey | 2001 | 515 | 0.63 (0.37) | 18.75 (18-20) | 65.44 | W | C | AD |
| ^a^McKinney et al. | 2008 | 475 | 0.60 (0.66) | 19.15 (18-25) | 68.2 | W | C | AD |
| ^b^McMahon et al. | 2002 | 209 | 0.27 | - (10-15) | 61.7 | W | C | D |
| ^b^Menon et al. | 2019 | 296 | 0.43 | 12.73 (10-15) | 43.9 | E | C | D |
| ^a^Miconi et al. | 2017 | 686 | 0.48 | 16.55 (14-20) | 43.7 | W | C | D |
| ^a^Miyamoto et al. | 2001 | 518 | 0.56 (0.51) | 15.6 (14-18) | 54.7 | W | C | AD |
| ^a^Moksnes et al. | 2012 | 1183 | 0.47 (0.60) | - (13-18) | 51 | W | C | AD |
| ^a^Moksnes et al. | 2016 | 1239 | 0.57 | 15 (-) | 51.2 | W | C | D |
| ^a^Moon | 2005 | 251 | 0.50 | 16.81 (-) | 60.6 | E | C | D |
| Author(s) | Year | N | *r* | M_age_ (age range) | Female | Culture | Design | Outcomes |
| ^a^Mosewich et al. | 2011 | 151 | (0.52) | 15.1 (-) | 100 | W | C | A |
| ^b^Muris et al | 2017 | 184 | (0.69) | 13.64 (12-16) | 54.3 | W | C | A |
| ^b^Neiss et al. | 2009 | 706 | 0.31 | 14.77 (-) | 100 | W | C | D |
| ^b^Nepon et al., | 2020 | 1039 | 0.60 | 15.2 (14-18) | 54.86 | W | L | D |
| ^b^Ngo et al. | 2020 | 89 | 0.37 | 16.3 (14-18) | 70 | W | L | D |
| ^a^Nguyen et al. | 2019 | 1149 | 0.43 (0.22) | - (16-18) | - | E | C | AD |
| ^b^O'Dea | 2009 | 470 | 0.19 | - (11-14.5) | 63.19 | W | C | D |
| ^a^O'Garo et al. | 2019 | 222 | 0.54 | 20.25 (-) | 70.9 | E | C | D |
| ^a^Oakley | 2001 | 161 | 0.70 | 16.6 (16-17) | 100 | W | C | D |
| ^b^Oliver et al. | 1995 | 186 | 0.66 | 19.5 (17-25) | 65.05 | W | C | D |
| ^b^Otterpohl et al. | 2019 | 211 | 0.53 | 13.66 (10-16) | 58.3 | W | C | D |
| ^a^Pan et al. | 2016 | 1506 | 0.68 | 15.21 (10-20) | 49.8 | E | C | D |
| ^a^Pantusa et al. | 2006 | 594 | 0.63 | 15.4 (11-18) | 50 | W | C | D |
| ^a^Park | 2000 | 259 | 0.54 | 15.23 (12-18) | 51.4 | W | C | D |
| ^a^Park | 2009 | 260 | 0.55 | 15.23 (12-18) | 51.4 | W | C | D |
| ^a^Park et al. | 2016 | 646 | 0.37 | 13.92 (12-15) | 44.9 | E | C | D |
| ^b^Parsi et al. | 2007 | 140 | 0.56 | 12.65 (12-15) | 62.1 | W | C | D |
| ^b^Pastor | 2020 | 610 | 0.39 | 13.38 (11-16) | 48 | W | C | D |
| ^a^Patterson | 2006 | 160 | 0.61 | 19.83 (18-25) | 61.9 | W | C | D |
| ^a^Paxton et al | 2006 | 2516 | 0.54 | 12.7 (-) | 54.6 | W | L | D |
| ^a^Piko et al. | 2003 | 1226 | 0.29 | 15.6 (11-20) | 47 | W | C | D |
| ^a^Piña-Watson et al. | 2019 | 722 | 0.45 | 19.69 (14-25) | 65.9 | W | C | D |
| ^a^Plunkett et al. | 2007 | 293 | 0.60 | 14.82 (14-17) | 57 | W | C | D |
| ^b^Porter | 2008 | 93 | 0.63 | 13.35 (11-17) | 68 | W | C | D |
| ^b^Prinstein et al. | 2002 | 246 | 0.59 (0.41) | 16.77 (15-18) | 60.16 | W | L | AD |
| ^a^Puckett | 2010 | 371 | 0.52 | 13.95 (13-15) | 57.95 | W | L | D |
| ^b^Quatman et al | 2001 | 380 | 0.41 | - (12-18) | 51 | W | C | D |
| Author(s) | Year | N | *r* | M_age_ (age range) | Female | Culture | Design | Outcomes |
| ^b^Ramey et al. | 2009 | 5015 | 0.39 | 15.77 (-) | 50 | W | C | D |
| ^a^Ranøyen et al. | 2015 | 5732 | 0.57 (0.64) | 15.8 (13-18) | 50.3 | W | C | AD |
| ^b^Rawana and Morgan | 2014 | 4359 | 0.57 | - (12-21) | 48.7 | W | L | D |
| ^a^Renk et al | 2006 | 116 | 0.74 | 19.79 (18-22) | 100 | W | C | D |
| ^a^Reuger et al. | 2017 | 491 | 0.63 | 13.2 (-) | 52 | W | L | D |
| ^b^Rhodes et al. | 2003 | 2585 | 0.59 | 12 (11-13) | 50 | W | L | D |
| ^a^Roberts & Gamble | 2001 | 153 | 0.34 | 16.1 (14-18) | 55 | W | C | D |
| ^b^Robins RW et al. | 2009 | 646 | 0.07 | 10.4 (-) | 49.7 | W | C | D |
| ^a^Robinson | 2009 | 955 | 0.71 | 19.7 (18-21) | 82 | W | C | D |
| ^a^Romera et al. | 2016 | 2,060 | (0.33) | 14.34 (12-19) | 47.9 | W | C | A |
| ^b^Rouquette et al. | 2021 | 124 | (0.44) | 16.23 (-) | - | W | C | A |
| ^b^Reuger et al. | 2009 | 636 | 0.55 (0.33) | 13.5 (12-15) | 51 | W | L | AD |
| ^a^Sainio et al | 2013 | 4941 | 0.55 | 14.41 (14-15) | 52.3 | W | L | D |
| ^b^Saint-Georges and Vaillancourt | 2020 | 612 | 0.72 | 12.42 (12-13) | 54 | W | L | D |
| ^a^Sakellari et al. | 2017 | 193 | 0.19 | 21.2 (-) | 66.32 | E | C | D |
| ^b^Salem | 2004 | 164 | 0.63 | 11.7 (11-13) | 55.49 | W | C | D |
| ^a^Sapouna et al. | 2013 | 3136 | 0.19 | - (13-14) | 51.5 | W | L | D |
| ^a^Sarkova et al. | 2013 | 1023 | 0.36 | 14.9 (14-17) | 52.4 | W | C | D |
| ^a^Schwartz et al. | 2017 | 302 | 0.43 | 14.51 (14-17) | 47 | W | L | D |
| ^a^Seaton | 2009 | 322 | 0.59 | 16 (13-18) | 53 | W | C | D |
| ^a^Seaton | 2010 | 322 | 0.59 | 16 (13-18) | 53 | W | C | D |
| ^a^Seaton et al | 2010 | 1170 | 0.52 | 15 (13-17) | 51.7 | W | C | D |
| ^b^Sheehan et al | 2002 | 152 | (0.64) | 16 (15-18) | - | W | C | A |
| ^a^Shen et al. | 2019 | 572 | 0.43 | - (18-25) | - | E | C | D |
| ^b^Siyez | 2008 | 1734 | 0.33 | - (14-18) | 53.72 | E | C | D |
| ^a^Smokowski | 2015 | 2617 | 0.35 (0.30) | 13.7 (-) | 54.49 | W | L | AD |
| Author(s) | Year | N | *r* | M_age_ (age range) | Female | Culture | Design | Outcomes |
| ^a^Smokowski et al | 2009 | 281 | (0.64) | 15 (11-19) | 55 | W | L | A |
| ^a^Son et al. | 2021 | 166 | 0.54 | 13.79 (11-16) | 59.6 | E | C | D |
| ^a^Southall et al. | 2002 | 115 | 0.69 | 16.5 (14-19) | 50.43 | W | L | D |
| ^a^Stebbins et al | 2009 | 510 | 0.75 | 13.68 (11-18) | 48.4 | W | C | D |
| ^a^Stolow et al. | 2016 | 193 | 0.63 | 13 (-) | 59 | W | L | D |
| ^a^Sze | 2016 | 911 | 0.05 (0.45) | 13.24 (-) | 49 | W | C | AD |
| ^a^Tan et al. | 2012 | 106 | 0.18 (0.01) | - (-) | - | W | C | AD |
| ^b^Taylor et al | 2013 | 326 | 0.32 (0.29) | 12.1 (10-16) | 53.7 | W | L | AD |
| ^a^Thøgersen-Ntoumani C et al. | 2011 | 93 | 0.44 (0.47) | 19.4 (-) | 100 | W | C | AD |
| ^a^Tian et al. | 2020 | 1047 | 0.52 | 13.54 (-) | 58.45 | E | L | D |
| ^a^Tian et al. | 2020 | 1200 | 0.39 | 12.63 (-) | 51.83 | E | L | D |
| ^a^Tomlinson | 2005 | 108 | 0.76 | 14.35 (12-17) | 100 | W | C | D |
| ^b^Trigueros et al. | 2019 | 547 | (0.81) | 17.14 (16-19) | 47.17 | W | C | A |
| ^b^Tu | 2017 | 106 | 0.63 (0.57) | 13.61 (-) | 55 | W | C | AD |
| ^a^Tuijl et al. | 2014 | 1641 | 0.59 (0.64) | 13.14 (10-16) | 54.3 | W | L | AD |
| ^a^Ubertini | 2010 | 163 | 0.67 (0.5) | 14.72 (13-18) | 77.9 | W | C | AD |
| ^a^Valentiner et al. | 2011 | 317 | 0.60 (0.57) | 19 (-) | 60.9 | W | C | AD |
| ^b^Vazquez-Garcia | 2004 | 117 | 0.28 | - (15-16) | 51.28 | W | C | D |
| ^b^Verhagen et al. | 2018 | 1342 | 0.36 | 13.94 (-) | 51 | W | C | D |
| ^a^Veselska et al. | 2009 | 3725 | 0.47 (0.47) | 14.3 (11-17) | 51 | W | C | AD |
| ^b^Voll | 2012 | 914 | (0.50) | - (11-14) | 52.8 | W | C | A |
| ^a^Wang et al. | 2016 | 589 | 0.28 | 15.76 (12-19) | 57.05 | W | C | D |
| ^a^Wang et al. | 2020 | 921 | 0.16 (0.35) | 12.98 (12-15) | 51.7 | E | L | AD |
| ^a^Wang et al. | 2020 | 2407 | 0.58 | 12.75 (11-16) | 49.9 | E | C | D |
| ^a^Wasylkiw et al. | 2012 | 142 | 0.71 | 19 (17-22) | 100 | W | C | D |
| ^a^Weber et al. | 2010 | 179 | 0.70 | 15.6 (14-18) | 52.5 | W | C | D |
| ^a^Wei | 2018 | 612 | (0.29) | 15.84 (15-16) | 55 | E | L | A |
| Author(s) | Year | N | *r* | M_age_ (age range) | Female | Culture | Design | Outcomes |
| ^b^Wilcox | 2003 | 244 | 0.33 (0.29) | - (12-14) | 50.82 | W | C | AD |
| ^b^Wouters et al. | 2016 | 599 | 0.14 | 17.7 (15-22) | 51 | W | C | D |
| ^a^Wouters et al. | 2018 | 1958 | 0.43 | 15.31 (-) | 56.28 | W | C | D |
| ^a^Wright | 2005 | 289 | 0.62 | 14 (-) | 53.3 | W | C | D |
| ^a^Yahav et al. | 2008 | 255 | (0.24) | 14.7 (14-16) | - | E | C | A |
| ^a^Yarcheski et al. | 2000 | 225 | 0.43 (0.60) | 12.4 (12-14) | 56 | W | C | AD |
| ^b^Yeh et al. | 2012 | 128 | 0.27 | 15.15 (11-20) | 46.9 | W | C | D |
| ^a^Yong-Rae et al. | 2020 | 251 | 0.58 (0.43) | 19.4 (-) | 31.1 | E | C | AD |
| ^a^Yoon et al | 2019 | 404 | 0.28 | - (17-19) | 44 | W | L | D |
| ^a^Yu et al. | 2014 | 455 | 0.67 | 19.86 (18-21) | 49.2 | E | C | D |
| ^a^Zhou et al. | 2020 | 1009 | 0.59 (0.37) | 12.97 (10-15) | 50.7 | E | L | AD |

*Note. ^a^* = Rosenberg Self-Esteem Scale (Rosenberg, 1965); ^b^ = other scales such as Contingencies of Self-Worth Scale (Crocker et al. 2003) and Collective Self-Esteem Scale Revised (Luhtanen & Crocker, 1992); *r* = effect size (in parentheses refer to anxiety) ; N = sample size; Female = percentage of adolescent sample who are female; Design = study design (C = cross-sectional; L = longitudinal; R = Randomised Controlled Trial); Culture = sample’s cultural background (E = Eastern; W = Western); Outcomes (A = Anxiety only; D = Depression only; AD = Anxiety and Depression)

**Table A3**

*Characteristics of Studies on Adolescent Self-Concept and Mental Health Included in the Meta-Analyses*

| Author(s) | Year | N | *r* | M_age_ (age range) | Female | Culture | Design | Outcomes |
| --- | --- | --- | --- | --- | --- | --- | --- | --- |
| ^a^Burns | 2013 | 180 | 0.57 (0.45) | - (10-12) | 57.77 | W | C | AD |
| ^d^Burwell and Shirk | 2006 | 110 | 0.35 | 13.62 (12-15) | 58.2 | W | L | D |
| ^d^Cheung | 2006 | 1043 | 0.47 | 16.04 (-) | 38.7 | E | C | D |
| ^d^Crocker et al. | 2006 | 501 | (0.35) | - (14-15) | 100 | W | L | A |
| ^c^Fiorilli et al. | 2019 | 182 | 0.38 | 12.7 (10-14) | 50.5 | W | C | D |
| ^d^Gadbois et al. | 2010 | 209 | (0.26) | - (-) | 73 | W | C | A |
| ^d^Goldstein | 2016 | 1464 | (0.11) | 12.84 (11-15) | - | W | L | A |
| ^a^Grøholt | 2005 | 65 | 0.23 | 17 (13-19) | 90 | W | C | D |
| ^d^Issacs et al. | 2008 | 177 | 0.48 | - (14-15) | 57.1 | W | L | D |
| ^d^Khalaila | 2014 | 170 | (0.23) | 21 (18-35) | - | E | C | A |
| ^a^Kirsh | 2006 | 137 | 0.31 | 19.23 (17-24) | 68.2 | W | C | D |
| ^a^Massey | 2001 | 515 | 0.63 (0.70) | 18.75 (18-20) | 65.44 | W | C | AD |
| ^a^Muris et al | 2017 | 184 | (0.19) | 13.64 (12-16) | 54.3 | W | C | A |
| ^d^Muris et al. | 2020 | 106 | 0.41 (0.38) | 13.65 (12-17) | 49 | W | C | AD |
| ^d^Murius et al. | 2015 | 132 | 0.67 (0.45) | 14.8 (12-17) | 57.58 | W | C | AD |
| ^d^Neiss et al. | 2009 | 706 | 0.31 | 14.77 (-) | 100 | W | C | D |
| ^b^Nepon et al. | 2020 | 1039 | 0.62 | 15.2 (14-18) | 54.86 | W | L | D |
| ^a^O'Dea | 2009 | 470 | 0.19 | - (11-14.5) | 63.19 | W | C | D |
| ^a^Pastor | 2020 | 610 | 0.39 | 13.38 (11-16) | 48 | W | C | D |
| ^c^Peresie | 2002 | 72 | 0.54 | 16.5 (15-19) | 72.22 | W | C | D |
| ^a^Prinstein et al. | 2002 | 246 | 0.36 (0.41) | 16.77 (15-18) | 60.16 | W | L | AD |
| ^b^Rawana and Morgan | 2014 | 4359 | 0.57 | - (12-21) | 48.7 | W | L | D |
| ^b^Robins RW et al. | 2009 | 646 | 0.07 | 10.4 (-) | 49.7 | W | C | D |
| ^b^Rouquette et al. | 2021 | 124 | (0.44) | 16.23 (-) | 0 | W | C | A |
| ^d^Santo et al. | 2018 | 141 | 0.33 | 10.99 (10-13) | 52.48 | W | L | D |
| ^b^Trigueros et al. | 2019 | 547 | (0.34) | 17.14 (16-19) | 47.17 | W | C | A |
| ^a^Vannucci et al. | 2017 | 753 | 0.40 | 16.09 (15-17) | 52.9 | W | L | D |

*Note. ^a^* = Self-Perception Profile for Children (Harter, 1985); ^b^ = Self-description Questionnaire (Marsh, 1988); ^c^ = Multidimensional Self-Concept Scale (Bracken, 1992); ^d^ = other scales such as Self-Concept Clarity Scale (Campbell et al., 1996); *r* = effect size (in parentheses refer to anxiety); N = sample size; Female = percentage of adolescent sample who are female; Design = study design (C = cross-sectional; L = longitudinal; R = Randomised Controlled Trial); Culture = sample’s cultural background (E = Eastern; W = Western); Outcomes (A = Anxiety only; D = Depression only; AD = Anxiety and Depression)

**Table A4**

*Characteristics of Studies on Adolescent Self-Awareness and Mental Health Included in the Meta-Analyses*

| Author(s) | Year | N | *r* | M_age_ (age range) | Female | Culture | Design | Outcomes |
| --- | --- | --- | --- | --- | --- | --- | --- | --- |
| ^a^Adams | 2008 | 276 | 0.10 | 13.07 (11-15) | 57.97 | W | L | D |
| ^b^Au et al. | 2009 | 6340 | 0.34 | - (10-13) | 49.37 | E | C | D |
| ^b^Canas et al. | 2019 | 1318 | 0.21 | 13.8 (11-18) | 53 | W | C | D |
| ^b^Chang | 2001 | 268 | 0.51 | 15.77 (14-19) | 61.12 | W | C | D |
| ^b^DeRosa | 2000 | 132 | 0.20 | 15.58 (13-18) | 61.4 | W | C | D |
| ^b^Cortés-Denia | 2020 | 845 | 0.28 (0.28) | 18 (15-20) | 56 | E | C | AD |
| ^b^Erkolahti et al. | 2003 | 1054 | 0.68 | 14.5 (-) | 56 | W | C | D |
| ^b^Estévez et al. | 2020 | 1318 | 0.49 | 13.8 (11-18) | 53 | W | C | D |
| ^b^Fernandez-Berrocal et al. | 2006 | 250 | 0.41 | 14.7 (14-19) | 52 | W | C | D |
| ^b^Flett et al. | 2006 | 1250 | 0.34 | 17.66 (-) | 100 | W | C | D |
| ^b^Gill et al. | 2018 | 316 | 0.55 | 14.77 (14-18) | 46.2 | W | C | A |
| ^b^Hards et al. | 2019 | 822 | 0.33 | 14.85 (13-18) | 55 | W | C | D |
| ^b^Kagawa | 2008 | 126 | 0.43 | - (-) | 61 | W | C | D |
| ^b^Lee et al. | 2006 | 327 | 0.39 (0.39) | 16 (-) | 100 | W | C | AD |
| ^a^Lee et al. | 2018 | 481 | 0.64 | 14.39 (13-15) | - | E | C | D |
| ^b^Iovu et al. | 2016 | 1509 | 0.41 (0.31) | 14 (13-15) | 59 | W | L | AD |
| ^b^Masi et al. | 2000 | 150 | 0.19 | 15.6 (14-18) | 51.3 | W | C | D |
| ^b^Parsi et al. | 2007 | 140 | 0.56 | 12.65 (12-15) | 62.1 | W | C | D |
| ^b^Polak et al. | 2012 | 60 | 0.27 (0.29) | 16.5 (-) | 41.7 | W | C | AD |
| ^b^Pradhan et al | 2007 | 131 | 0.51 (0.42) | 17 (14-20) | 52 | W | C | AD |
| ^b^Prinstein et al. | 2002 | 246 | 0.40 (0.29) | 16.77 (15-18) | 60.16 | W | L | AD |
| ^b^Santo et al. | 2018 | 141 | 0.43 | 10.99 (10-13) | 52.48 | W | L | D |
| ^a^Sebokova et al. | 2016 | 294 | 0.12 | 17.35 (14-21) | 63.61 | W | C | A |
| ^b^Son et al. | 2021 | 166 | 0.59 | 13.79 (11-16) | 59.6 | E | C | D |
| ^b^Schwartz et al. | 2006 | 167 | 0.79 | 12.39 (12-14) | 39 | W | C | D |
| ^b^Tenaglia | 2020 | 27 | 0.26 (0.19) | - (14-18) | 70.4 | W | C | AD |
| Author(s) | Year | N | *r* | M_age_ (age range) | Female | Culture | Design | Outcomes |
| ^b^Wang | 2011 | 1171 | 0.31 (0.67) | 12.2 (10-16) | 53.2 | W | L | AD |
| ^b^Wang et al | 2017 | 365 | 0.28 | 15.96 (14-18) | 52 | E | C | D |
| ^b^Xu et al. | 2019 | 411 | 0.46 (0.32) | 19.55 (17-24) | 68.1 | E | C | AD |
| ^b^Weinman et al. | 2003 | 110 | 0.57 | 19 (13-22) | 95 | W | C | D |
| ^a^Wright | 2005 | 289 | 0.62 | 14 (-) | 53.3 | W | C | D |
| ^d^Zaki et al. | 2019 | 200 | 0.30 | 13.73 (14-16) | 73 | E | C | D |
| ^b^Zhang et al. | 2013 | 1297 | 0.18 | 13.9 (13-14) | 47.3 | E | C | D |

*Note. ^a^* = Self-consciousness Scale (Fenigstein et al., 1975); ^b^ = other scales such as Objectified Body Consciousness Scale (OBCS) (McKinley and Hyde 1996); *r* = effect size (in parentheses refer to anxiety); N = sample size; Female = percentage of adolescent sample who are female; Design = study design (C = cross-sectional; L = longitudinal; R = Randomised Controlled Trial); Culture = sample’s cultural background (E = Eastern; W = Western); Outcomes (A = Anxiety only; D = Depression only; AD = Anxiety and Depression)

**Table A5**

*Characteristics of Studies on Adolescent Self-Efficacy and Mental Health Included in the Meta-Analyses*

| Author(s) | Year | N | *r* | M_age_ | Female | Culture | Design | Outcomes |
| --- | --- | --- | --- | --- | --- | --- | --- | --- |
| ^b^Bandura et al. | 2003 | 464 | 0.25 | 16 (14-19) | 54.09 | W | L | D |
| ^b^Caprara et al. | 2010 | 452 | 0.28 | 12.81 (11-13) | 50.22 | W | L | D |
| ^b^Carter et al. | 2020 | 559 | 0.36 | 14.99 (13-17) | - | W | C | D |
| ^b^Dou et al. | 2016 | 1108 | 0.45 | 14.65 (11-17) | 50.63 | E | C | D |
| ^b^Flett et al. | 2021 | 253 | 0.12 | 19.8 (-) | 67.6 | W | C | D |
| ^a^Flett et al. | 2021 | 134 | 0.34 | 17 (-) | 53.7 | W | C | D |
| ^b^Gadbois et al. | 2010 | 209 | (0.40) | - (-) | 73 | W | C | A |
| ^b^Galand et al. | 2013 | 406 | 0.59 | 13 (11-16) | 47 | W | C | D |
| ^b^Garaigordobil | 2015 | 286 | 0.21 | 14.72 (14-16) | 48.3 | W | C | D |
| ^a^Loton et al. | 2017 | 11138 | 0.47 (0.34) | 14.04 (10-18) | 41 | W | C | AD |
| ^b^Murius et al | 2017 | 184 | (0.52) | 13.64 (12-16) | 54.3 | W | C | A |
| ^b^Murius et al. | 2015 | 132 | 0.53 (0.58) | 14.8 (12-17) | 57.58 | W | C | AD |
| ^b^Oliver et al. | 1995 | 186 | 0.33 | 19.5 (17-25) | 65.05 | W | C | D |
| ^b^Romera et al. | 2016 | 2,060 | (0.32) | 14.34 (12-19) | 47.9 | W | C | A |
| ^b^Smith and Betz | 2002 | 405 | 0.30 | 18.8 (-) | 68.14 | W | C | D |
| ^a^Tchakmakjian | 2003 | 126 | 0.42 (0.29) | 21.13 (18-24) | 47.6 | W | C | AD |
| ^b^Tyser | 2014 | 164 | 0.34 | 14 (-) | 53 | W | C | D |
| ^a^Vazquez-Garcia | 2004 | 117 | 0.27 | - (15-16) | 51.28 | W | C | D |
| ^b^Weinman et al. | 2003 | 110 | 0.52 | 19 (13-22) | 94.5 | W | C | D |
| ^b^Xu et al | 2017 | 416 | 0.35 | 16.13 (15-18) | 58.4 | E | C | A |
| ^b^Zhang et al. | 2013 | 1297 | 0.15 | 13.9 (13-14) | 47.3 | E | C | D |

*Note. ^a^* = General Self-Efficacy Scale (Schwarzer & Jerusalem, 1995); ^b^ = other scales such as Scale of Perceived Social Self-Efficacy (PSSE; Smith & Betz, 2000); *r* = effect size (in parentheses refer to anxiety); N = sample size; Female = percentage of adolescent sample who are female; Design = study design (C = cross-sectional; L = longitudinal; R = Randomised Controlled Trial); Culture = sample’s cultural background (E = Eastern; W = Western); Outcomes (A = Anxiety only; D = Depression only; AD = Anxiety and Depression)

**Table A6**

*Characteristics of Studies on Adolescent Self-Regulation and Mental Health Included in the Meta-Analyses*

| Author(s) | Year | N | *r* | M_age_ | Female | Culture | Design | Outcomes |
| --- | --- | --- | --- | --- | --- | --- | --- | --- |
| Cano et al. | 2020 | 200 | 0.21 | 21.3 (18-25) | 51 | W | C | D |
| Caprara et al. | 2010 | 452 | 0.15 | 12.81 (11-13) | 50.22 | W | L | D |
| Coyne et al. | 2019 | 385 | 0.48 (0.36) | 18.01 (17-19) | 53 | W | L | AD |
| Feld | 2014 | 661 | 0.10 | - (10-12) | 49.8 | W | L | D |
| Ferrari | 2008 | 246 | 0.21 | 15.1 (14-16) | 100 | W | C | A |
| Finkenauer et al. | 2005 | 1359 | 0.35 | 12.3 (10-14) | 47.8 | W | C | D |
| Gadbois et al. | 2010 | 209 | 0.60 | - (-) | 73 | W | C | A |
| Knoble | 2016 | 593 | 0.39 | 11.9 (11-12) | 49 | W | L | D |
| Moilanen and Manuel | 2019 | 302 | 0.32 | 21.57 (18-24) | 64.9 | W | C | D |
| Peresie | 2002 | 72 | 0.49 | 16.5 (15-19) | 72.22 | W | C | D |
| Rhodes et al. | 2013 | 387 | 0.41 | 12.1 (11-13) | 55 | W | L | D |
| Rudolph et al. | 2001 | 329 | 0.45 | 11.2 (10-12) | 51.1 | W | L | D |
| Stormshak | 2018 | 593 | 0.27 | 11.83 (-) | 49 | W | R | D |
| Trigueros et al. | 2019 | 547 | 0.19 | 17.14 (16-19) | 47.17 | W | C | A |
| Weinman et al. | 2003 | 110 | 0.31 | 19 (13-22) | 94.5 | W | C | D |

*Note. r* = effect size (in parentheses refer to anxiety); N = sample size; Female = percentage of adolescent sample who are female; Design = study design (C = cross-sectional; L = longitudinal; R = Randomised Controlled Trial); Culture = sample’s cultural background (E = Eastern; W = Western); Outcomes (A = Anxiety only; D = Depression only; AD = Anxiety and Depression)

**Table A7**

*Characteristics of Studies on Adolescent Self-Compassion and Mental Health Included in the Meta-Analyses*

| Author(s) | Year | N | *r* | M_age_ | Female | Culture | Design | Outcomes |
| --- | --- | --- | --- | --- | --- | --- | --- | --- |
| ^a^Baldini | 2018 | 11 | 0.77 (0.60) | 15.5 (14-18) | 54.5 | W | R | AD |
| ^a^Barry et al. | 2015 | 251 | 0.27 (0.32) | 16.78 (16-18) | 0 | W | C | AD |
| ^a^Barutçu Yıldırım and Demir | 2020 | 801 | (0.42) | NA (18-25) | 50.4 | E | C | A |
| ^b^Bluth et al. | 2017 | 765 | (0.38) | NA (11-19) | 53 | W | C | A |
| ^a^Budakoglu et al. | 2014 | 225 | (0.06) | 19.4 (17-25) | 54.2 | E | C | A |
| ^a^Castilho et al. | 2017 | 1101 | 0.62 | 15.94 (NA) | 57.4 | W | C | D |
| ^a^Chu et al. | 2018 | 489 | 0.51 (0.40) | 12.67 (11-15) | 43.6 | E | C | AD |
| ^a^Cunha et al. | 2013 | 651 | 0.46 (0.33) | 15.89 (12-18) | 49.3 | W | C | AD |
| ^a^Cunha et al. | 2016 | 3165 | 0.38 (0.29) | 15.49 (12-19) | 53.8 | W | C | AD |
| ^a^Gill et al. | 2018 | 316 | (0.55) | 14.77 (14-18) | 46.2 | W | C | A |
| ^a^Hou et al. | 2020 | 578 | 0.43 | 20.3 (17-24) | 47.8 | E | C | D |
| ^b^Jiang et al. | 2020 | 728 | 0.27 | 14.07 (12-16) | 51.1 | E | C | D |
| ^b^Kishida et al. | 2004 | 574 | 0.34 (0.43) | 20.1 (NA) | 74.56 | E | C | AD |
| ^a^Ko et al. | 2018 | 41 | 0.59 (0.52) | 19.78 (18-22) | 66 | W | R | AD |
| ^a^Lahtinen et al. | 2019 | 2383 | 0.32 | NA (16-18) | 52.4 | W | C | D |
| ^a^Lathren et al. | 2019 | 1057 | 0.62 | 14.7 (12-18) | 65 | W | C | D |
| ^a^Liu | 2020 | 871 | (0.40) | 15.18 (NA) | 45.3 | E | C | A |
| ^a^Liu et al. | 2020 | 1020 | 0.52 | 16.82 (15-19) | 46.08 | E | C | D |
| ^a^Mosewich et al. | 2011 | 151 | (0.37) | 15.1 (NA) | 100 | W | C | A |
| ^a^Muris et al | 2017 | 184 | (0.19) | 13.64 (12-16) | 54.3 | W | C | A |
| ^a^Muris et al. | 2018 | 130 | 0.43 (0.38) | 13.65 (12-17) | 66.2 | W | C | AD |
| ^a^Muris et al. | 2020 | 106 | 0.41 (0.49) | 16.68 (NA) | 49 | W | C | AD |
| ^a^Murius et al. | 2015 | 132 | 0.30 (0.26) | 14.8 (12-17) | 57.58 | W | C | AD |
| Author(s) | Year | N | *r* | M_age_ | Female | Culture | Design | Outcomes |
| ^a^Neff and McGehee | 2010 | 522 | 15.2 (14-17) | 15.2 (14-17) | 52 | W | C | AD |
| ^a^Raymond | 2018 | 172 | 19.2 (-) | 20 (18-33) | 81.8 | W | C | A |
| ^a^Stefan | 2019 | 63 | 18.76 (-) | 19.2 NA) | 75 | W | L | A |
| ^a^Stefan et al. | 2020 | 110 | 18.88 (-) | 18.76 (NA) | 85 | W | C | A |
| ^a^Stolow et al. | 2016 | 193 | 13 (-) | 18.88 (NA) | 59 | W | L | D |
| ^a^Tanaka et al. | 2011 | 561 | 15.8 (14-17) | 13 (NA) | 53 | W | L | D |
| ^a^Temel et al. | 2018 | 529 | 14.96 (14-17) | 15.8 (14-17) | 42.72 | E | C | AD |
| ^a^Wasylkiw et al. | 2012 | 142 | 19 (17-22) | 14.96 (14-17) | 100 | W | C | D |
| ^a^Wu et al. | 2019 | 813 | 13.15 (11-16) | 19 (17-22) | 43.3 | E | L | D |
| ^a^Xavier et al. | 2016 | 643 | 15.24 (12-18) | 13.15 (11-16) | 51.6 | W | C | D |

*Note. ^a^* = Self-Compassion Scale (Neff, 2003); ^b^ = other scales such as Self-Handicapping Scale (SHS; Jones and Rhodewalt, 1982); *r* = effect size (in parentheses refer to anxiety); N = sample size; Female = percentage of adolescent sample who are female; Design = study design (C = cross-sectional; L = longitudinal; R = Randomised Controlled Trial); Culture = sample’s cultural background (E = Eastern; W = Western); Outcomes (A = Anxiety only; D = Depression only; AD = Anxiety and Depression)
